# Dynamical Binding Modes Determine Agonistic and Antagonistic Ligand Effects in the Prostate-Specific G-Protein Coupled Receptor (PSGR)

**DOI:** 10.1038/s41598-017-16001-4

**Published:** 2017-11-22

**Authors:** Steffen Wolf, Nikolina Jovancevic, Lian Gelis, Sebastian Pietsch, Hanns Hatt, Klaus Gerwert

**Affiliations:** 1 Department of Biophysics, ND 04 North, Ruhr-University Bochum, 44780 Bochum, Germany; 2 Department of Biophysics, CAS-MPG Partner Institute for Computational Biology, Key Laboratory of Computational Biology, Shanghai Institutes for Biological Sciences, Chinese Academy of Sciences, 200031 Shanghai, P.R. China; 3 Department of Cellphysiology, ND 4, Ruhr-University Bochum, 44780 Bochum, Germany

## Abstract

We analysed the ligand-based activation mechanism of the prostate-specific G-protein coupled receptor (PSGR), which is an olfactory receptor that mediates cellular growth in prostate cancer cells. Furthermore, it is an olfactory receptor with a known chemically near identic antagonist/agonist pair, α- and β-ionone. Using a combined theoretical and experimental approach, we propose that this receptor is activated by a ligand-induced rearrangement of a protein-internal hydrogen bond network. Surprisingly, this rearrangement is not induced by interaction of the ligand with the network, but by dynamic van der Waals contacts of the ligand with the involved amino acid side chains, altering their conformations and intraprotein connectivity. Ligand recognition in this GPCR is therefore highly stereo selective, but seemingly lacks any ligand recognition via polar contacts. A putative olfactory receptor-based drug design scheme will have to take this unique mode of protein/ligand action into account.

## Introduction

Olfactory receptors (ORs), which are part of the G protein-coupled receptor (GPCR) family^1^, recently turned out to be present not only in the human nose, but in a large set of different human tissues as well^2,3^. A functional role of these ectopically expressed ORs was shown in human colon tissue^4^, sperm^5^, blood cells^6^, and skin tissue^7,8^. Furthermore, the expression of some ORs is up regulated in different types of cancer cells^9–13^, and these ORs have an effect on cell proliferation in at least prostate^2^, liver^14^, leukemia^15^ cancer and melanoma^16^ cells. One of these cancer-related receptors is OR51E2, known as well as the prostate-specific G protein-coupled receptor (PSGR) as this GPCR was first detected in the prostate before it was classified as an olfactory receptor by sequence homology^10,17^. Recent studies indicate that PSGR expression is up-regulated in prostate cancer^2^ and melanoma cells^16^, and that PSGR activation leads to an inhibition of cell proliferation^2,16,18,19^. Human melanoma is an aggressive and highly metastatic type of cancer, which is highly resistant to conventional therapies^20^. Prostate cancer is the 2^nd^ most common cause of cancer, and the 6^th^ leading cause of cancer death in men^21^. PSGR may therefore be a new target for the diagnosis and therapy of cancer, especially for notoriously difficult to treat melanoma.

To be able to design molecular therapeutics specific for PSGR, the prerequisites of molecular pharmacology for ORs need to be known. It was recently shown that though ORs are classified as rhodopsin-like GPCRs, they do not belong to the common rhodopsin-like small molecule binding GPCRs, but rather form a subclass of their own^22^. Consequently, OR ligand (odorant) recognition exhibits peculiar activation properties: each single OR can be activated by a large number of ligands, which translates into a cytosolic G protein response level that depends on the respective ligand bound^23^. Contrary to the seemingly unspecific ligand recognition, ORs can show remarkable ligand specificity: in earlier experimental studies, we established β-ionone as agonist for PSGR, and α-ionone as competitive antagonist^2,8,16^. PSGR and β-ionone/α-ionone are an OR agonist/antagonist combination with nearly identical ligand chemical scaffolds, i.e., they only differ in the position of a single carbon/hydrogen bond, and thus present a unique case to assess differences in OR/ligand interactions between these pharmacological subclasses. Agonist/antagonist pairs with a lower grade of structural similarity, i.e., they contain similar structural features like ring moieties, polar groups, or hydrophobic tails at comparable positions, are known for several other receptors^24–29^. In addition to ionones, we identified steroid hormones as activators of PSGR^2,8^. However, as numerous GPCR crystal structures have shown^30^, cholesterol scaffold-based compounds do not bind to the center of the 7TM helix bundle, but to the protein/membrane interface at helices II and IV. Therefore, we hold these steroids to be allosteric activators of PSGR, and do not investigate them here further. The peculiar ligand recognition properties of olfactory receptors were proposed to be based on recognition by shape components (so-called “odotopes”)^31–35^, molecular vibrations^36^, or a combination of both^37^. In a pilot study on the olfactory receptor hOR2AG1, we established an alternative model, which is a combination of shape recognition and matching protein–ligand dynamics^38^. According to our “dynamical ligand binding” model, besides ligand affinity^39^, it is the frequency of receptor and ligand contacts occurrence, which is decisive for receptor activation. Recently, this concept was nicely reproduced for the case of odorant recognition in the mouse olfactory receptors mOR-EG and mOR-EV^40^, and is not restricted to ORs, but applies to other small-ligand binding GPCRs as well^41^.

**Figure 1:**
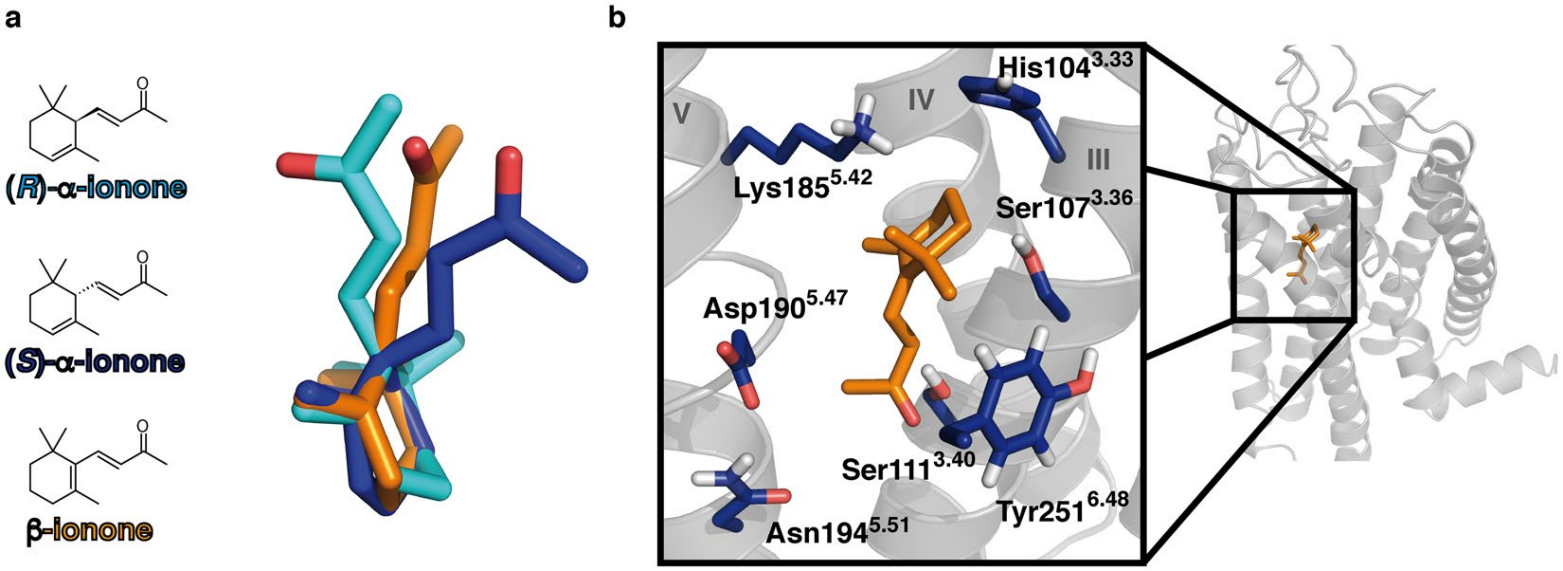
Structure of α-ionones and β-ionone, and the predicted ligand binding cavity. (*R*)- α-ionone in cyan, (*S*)- α-ionone in dark blue, β-ionone in orange. (**a**) Chemical structures and 3D structures of ionones after overlay of their ring moieties. The main difference between the three isoforms lies in the angle of the butenoneyl side chain to the ring moiety. (**b**) PSGR structural model with a close-up of the central binding site. Protein backbone in grey, β-ionone in its best docking posture in the inactive protein state in orange sticks, surrounding mutated residues in blue sticks. Helices numbered in roman numbers. The displayed structure is the initial inactive protein model used for ligand docking. Mutation of all displayed residues had an effect on ligand binding and protein activation (see Table 1). We therefore propose this position within the protein model as the β-ionone binding site.

Figure 1a shows a comparison of the two ionones: α- and β-ionone are constitutional isomers, which only differ in the position of one double bond. Structurally, this leads to a different angle at the connection between the ionone ring moiety and the butenoneyl side chain. The two substances thus only differ slightly in their side molecular structure, while being chemically nearly identical. However, this small difference is recognized by PSGR, and leads to the discrimination into agonist and antagonist. α-ionone actually exists as two enantiomers, (*R*)- and (*S*)- α-ionone, which will bind to the receptor with different affinities, and potentially will even belong to different pharmacological classes. This substance was applied as racemic mix in earlier experiments^2^, and separate measurements of the enantiomers are not possible: due to being a β-unsaturated ketone, keto-enol-tautomerism includes the δ-carbon atom, i.e., the asymmetric atom within α-ionones. Enantiomeric pure (*R*)- or (*S*)- α-ionone would therefore quickly racemize in aqueous solution. Due to this chemical limitation, it is not clear which of both enantiomers is the actual antagonist.

In this work, we investigate the different dynamic binding modes of α- and β-ionone in PSGR by employing a close combination of experimental and theoretical methods. By the creation of a dynamic homology model^38,41,42^ of PSGR in combination with ligand docking^41,43^, we propose a model of the binding site for both ligands within our OR of interest, and further analysed the different binding strengths of both enantiomers of α-ionone in comparison to β-ionone within the model. We then assessed their differences in dynamic binding modes of α-ionones in comparison to β-ionone by analysing the frequency of occurrence of their dynamic protein/ligand and protein/protein contacts in the form of dynamic interaction fingerprints (IFPs)^38,41,44,45^. For this, we build two homology models, based on a rhodopsin crystal structure in its inactive state^46^, and an opsin crystal structure in an activated state with a bound hydrophobic lipid molecule^47^ (see Figure S1 for the used sequence alignment). Though olfactory receptors form a separate subclass of GPCRs^22^, we chose rhodopsin as starting model, as it proved to be a good structural template in our earlier investigation on hOR2AG1^38^, and is the only GPCR that contains a hydrophobic ligand and is at the same time available as structural model in both an inactive and an active-like conformation. We verified our theoretical results by extensive comparison with experimental results on PSGR activation derived from luciferase reporter assays in the heterologous expression system in combination with point mutation analysis of recombinant receptors. Both theoretical and experimental results matched well, and allowed us to propose a dynamic binding mode and a dynamic protein/ligand and protein/protein IFP necessary for activation. To our surprise, this binding mode showed to be highly stereo selective, but not to include any polar contacts: The ionones were recognized by their overall shape, but no significant occurrence of a protein/ligand hydrogen bond via the keto moiety was observed. This matched well with an overall low affinity for ionones observed in experiments. A future development of OR-based drug design will need to take this mode of hydrophobic ligand action into account.

## Results

### Determination of the β-ionone binding site

To start our investigation, we needed to find a putative ionone-binding pocket within PSGR. For this, we focused on β-ionone, as it is the compound in our investigation, which induces protein activation, and thus results in a clear experimental output signal. As protein model, we used the inactive ligand free rhodopsin-based PSGR homology model, as this would be the structure β-ionone would need to bind to according to the ternary complex model^48^.

We here have to state that with a 20% sequence identity between PSGR and rhodopsin, the overall homology between target and template is relatively low, and that the cut-off for a “reliable” GPCR homology model is at a minimum of 35% identity, as pointed out by Kufareva *et al*.^49^. However, this is a general problem with modelling odorant receptors. The success of generating homology models of ORs based on crystal structures of rhodopsin and other GPCRs (see refs^33,38–40,50–52^) justifies homology modelling of ORs to construct molecular models. However, these models per se have predictive character, but cannot be taken as competitors of experimentally resolved structures. Nevertheless, comparison with experiment offers an additional source of data for model refinement: identifying models that integrate most of the experimental data can allow formulating hypotheses and predictions for the actual dynamics of the receptor and receptor activation. We have shown this successfully by reprogramming the selectivity filter of hOR2AG1 by predicting a point mutation that leads to activation of the receptor by a ligand that does not activate wild type protein^38^.

**Figure 2:**
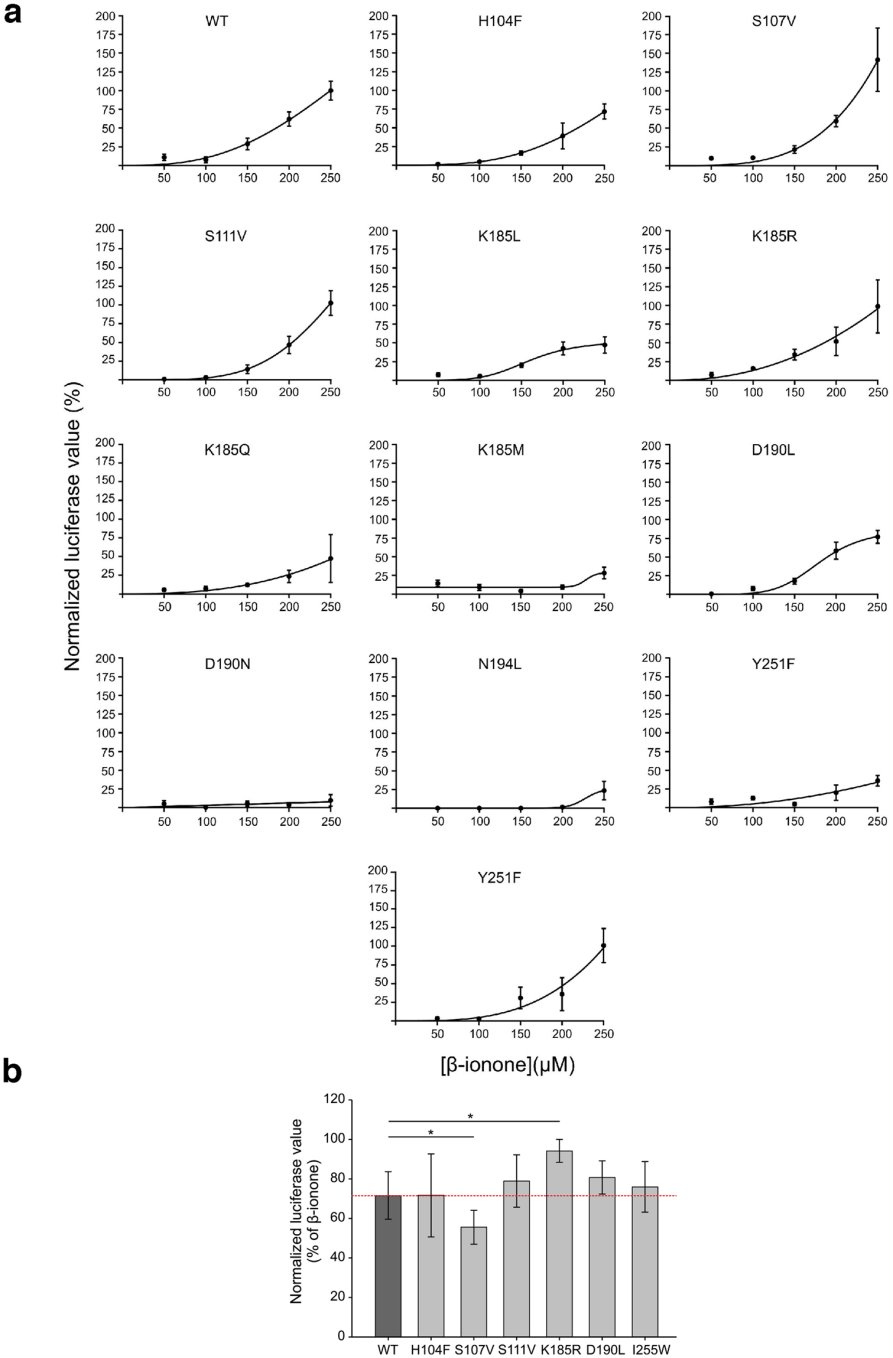
Dose-response curves of PSGR variants. (**a**) Responses of cells transfected either with a plasmid encoding for wild type (WT) and point-mutated PSGR, respectively, to β-ionone as measured by luciferase assay. Receptor activation was normalized to the relative response to β**-**ionone (250 µM) in WT transfected cells. Dose-response curves were fitted by a *Hill* equation. Odorant concentrations up to 250 µM were used because higher concentrations of β-ionone exhibited toxic effects on the cells as cells detached during treatment. Error bars indicate the standard error of the mean (SEM) of 4–15 replicas. (**b**) Quantification of luciferase values of Hana3a cells transiently expressing wild type (WT) or point-mutated PSGR co-stimulated with α-ionone (200 µM) and β-ionone (200 µM) or β-ionone (200 µM) only. The blocking effect of α-ionone was normalized to the corresponding response to β-ionone of the respective receptor. The data are shown as the means ± SEM (n = 3). Significance was calculated by Student’s t-test (*p < 0.05).

Figure 1b shows the putative β-ionone binding position appearing during docking runs. The site is positioned within the centre of the 7TM helix bundle between helices III, IV, V, and VI. It therefore is in good agreement with the position of the ionone ring of retinal within rhodopsin^46^ and earlier works on olfactory receptor binding sites^33,38–40,50–54^. Based on these results, we determined the amino acids constituting this site, and carried out functional analysis of point-mutated receptors in order to verify the binding site. These amino acids (see Fig. 1b; numbering in superscripts are the respective Ballesteros-Weinstein residue numbers^55^) were His104^3.33^, Ser107^3.36^, and Ser111^3.40^ in helix III; Lys185^5.42^, Asp190^5.47^, and Asn194^5.51^ in helix V; and Tyr251^6.48^ in helix VI. All mutant receptors were functionally expressed in Hana3A cells as controlled by live-cell immunocytochemical staining (see Supplementary Figure S2). Table 1, and Figs 2a and S3a show the effect of the respective mutations on the experimentally observable threshold concentration and the maximal receptor activation (E_max_). We here need to point out that we use threshold concentrations, and not EC_50_ values, as such EC_50_ values would have been observable at concentrations >250 µM β-ionone, which showed cytotoxic effects in our luciferase essays. Furthermore, there is a significant difference in β-ionone potencies determined in the study of Neuhaus *et al*. by single cell calcium imaging of transiently transfected HEK293 cells^2^ compared to our approach using Hana3a cells and the CRE-mediated luciferase assay, which may result from differential assessment of potencies or from biased signalling of PSGR. We therefore recommend to not to take the respective E_max_ and response curves as absolute values, but to consider them for a comparison of differences within the framework of this article. Mutation of each investigated residue showed an impact on both thresholds and E_max_. Interestingly, we observed not only receptor mutants with decreased thresholds/E_max_, but also neutral point mutations as well as mutants with increased thresholds/E_max_ (“hyperactive” mutants). We provide a detailed analysis of these mutants in the MD simulation section.

**Table 1:**
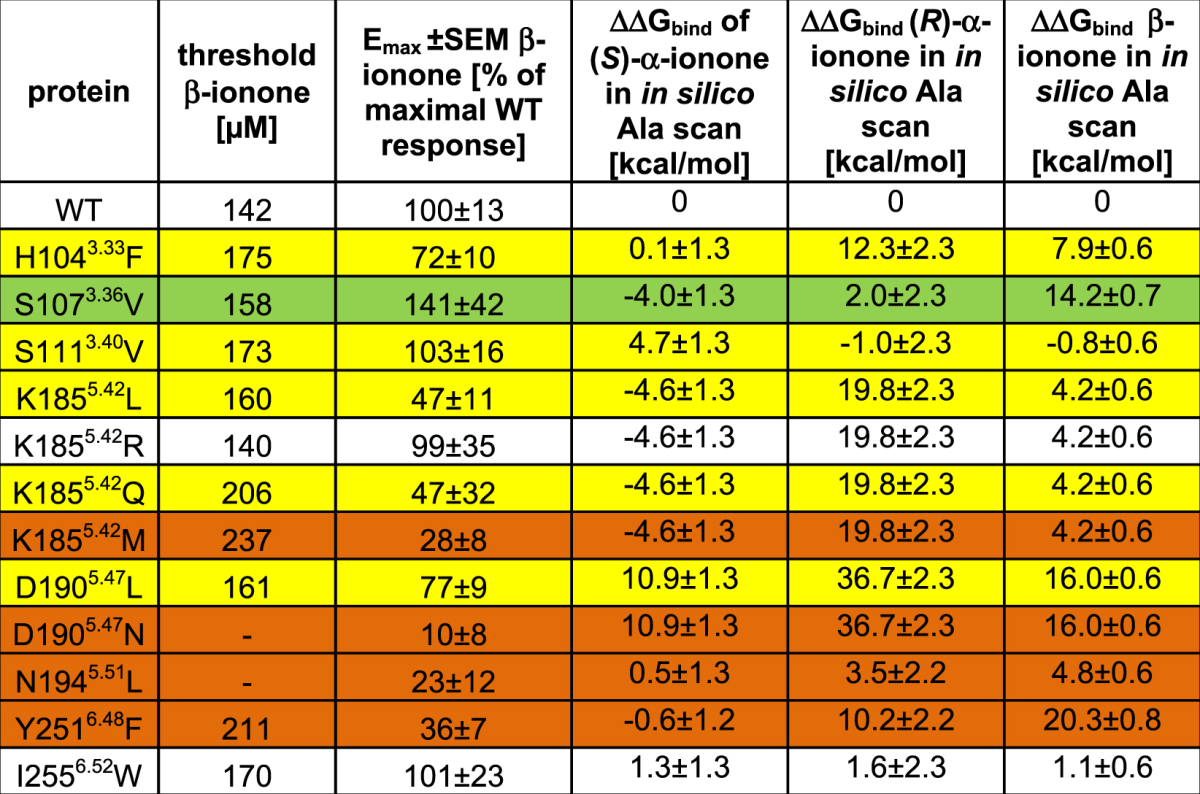
Effects of mutation of residues flanking the central binding site on ionone binding. (measured as threshold, i.e. a 10% signalling output level of 10 µM forskolin) and subsequent maximal receptor activation (E_max_, see Fig. S3 for details) ± SEM by 250 μM (normalized to wild type; n = 4–15). Colour coding: hyperactive mutants (E_max_ > 110% of WT) in green; unaffected mutants (threshold 170–140 µM and E_max_ 110–80% of WT) in white; affected mutants (threshold 210–170 µM and E_max_ 80–40% of WT) in yellow; inactive mutants (threshold >210 µM and E_max_ <40% of WT) in orange. A decrease in E_max_ is coupled to an increase in threshold concentration. Mutations to Ala *in silico* of the respective residues that lead to a significant change in ΔΔG_bind_ mostly corresponds to an alteration of activation in experiment, as well. The control mutant residue I255^6.52^ does not exhibit a significant influence on ligand binding.

As control, we introduced an I255^6.52^W mutation. This mutation targets a residue close to the proposed binding site, but the respective native side chain does not make a contact with the docked ligand, nor does it interfere with the neighbouring helices (see Supplementary Figure S4). As to be expected, the mutant exhibits a near-native threshold and E_max_, which further confirms our proposed protein homology model and the binding position for β-ionone.

As an additional check to correlate individual amino acids and receptor activation, we investigated if the mutants had an effect on the basal activity of the receptor^56,57^. The results are summarized in Table SI: we observed 2-8fold decreases in basal activities for the mutants K185^5.42^R, K185^5.42^Q, and D190^5.47^L, which further supports the involvement of these residues in receptor activation.

**Figure 3:**
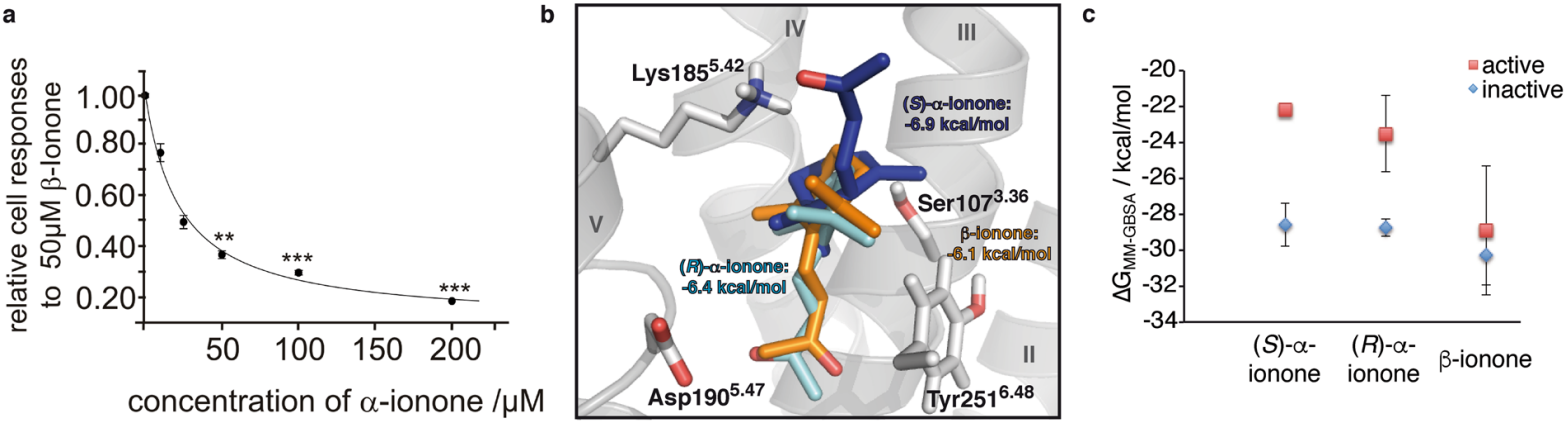
Experimental ligand competition between α-ionones, and β-ionone in comparison with predicted binding affinities from Docking and free energy calculations. (**a**) Experimental competition assay^2^. The β-ionone induced Ca^2+^ responses of HEK293 cells transiently expressing PSGR is inhibited by the co-application of α-ionone in a dose-dependent manner. Co-application of increasing concentrations of α-ionone caused an increased reduction of the number of cells responding to 50 μM β-ionone. Shown are relative numbers of cells responding to 50 μM β-ionone in the presence of varying concentrations of a racemic mix of α-ionones. Odorants were applied for 20 s. The data are the mean of 10 independent experiments for every α-ionone concentration tested, each with 600 to 1600 cells. Significance was calculated by Student’s t-test for each sample group referring to cell responses to β-ionone (50 µM). Error bars represent SEM, (*p < 0.05, **p < 0.01 and ***p < 0.001). α-ionone blocks cellular responses to β-ionone, with an affinity that is seemingly twofold higher than the one of β-ionone. (**b**) Best docking poses of (*R*)-, (*S*)- α-ionone, and β-ionone found in the initial ligand-free inactive PSGR model with respective calculated ∆G_bind_ values. Protein backbone in grey, β-ionone in orange sticks, (*R*)- α-ionone as cyan sticks, (*S*)- α-ionone as blue sticks. Selected surrounding protein residues in grey sticks. Helices numbered in roman numbers. β-ionone and (*R*)- α-ionone bind in a very similar fashion with the head groups oriented towards Asp190^5.47^; the detailed binding mode however differs, as (*R*)- α-ionone presents its keto group oxygen atom to Asp190^5.47^, while β-ionone presents the terminal methyl group to Asp190^5.47^. (*S*)- α-ionone binds with the head group oriented towards Lys185^5.42^. Both α-ionone enantiomers bind with a slightly higher affinity than β-ionone, which is in line with α-ionone being a competitive antagonist for β-ionone. (**c**) Results from free energy calculations (see Table SIII). Both α-ionone stereoisomers exhibit a clearly separated affinity for the active and the inactive receptor conformation, with the affinity for the inactive conformation being significantly higher than the one for the active conformation. β-ionone exhibits an affinity for the receptor, which is comparable to the one of α-ionones for the inactive receptor, but within the range of its standard deviation the same for the active and the inactive receptor.

We need to point out that a recent study of Sanz *et al*.^58^ found α-ionone to be an agonist of heterlogously in HEK293 cells expressed PSGR. However, this work used a small number of experiments (>3), and the relative fluorescence intensity in non-ratiometric measurments of a small number of cells (>20) as main observable. For the results displayed in Fig. 3a, we rely on ten individual experiments (each with 600 to 1600 cells), and the relative number of cells responding to an odorant stimulus above a certain fluorescence threshold from a large number of individual cells. Furthermore, Sanz *et al*. do not comment on the statistical significance of their data, which exhibit a large standard deviation^58^. We therefore believe that the experimental setup and the number of experiments of Sanz *et al*.^58^ are too small provide the same granularity of results as we did.

### Analysis of α-ionone binding and antagonistic effect

Our previous experimental investigations^2^ showed α- and β-ionone to be competitive antagonist and agonist, respectively. To understand the molecular basis of this inhibition, we measured the cellular response of HEK293 cells to 50 µM β-ionone at varying concentrations of α-ionone^2^ via Ca^2+^-imaging. Figure 3a displays the resulting inhibition curve: a β-ionone/α-ionone concentration ratio of 2:1 led to 50% relative cellular response, a ratio of 1:1 to 36% cellular response, a ratio of 1:2 to 32% response, and a ratio of 1:4 to 20% cellular response. From this observation, we conclude that β-ionone and α-ionone compete for the same binding site, and that α-ionone exhibits the 1-2 – fold affinity of β-ionone for the receptor. However, as Fig. 1 shows, α-ionone exists as two different enantiomers, and the experimental investigations are based on a racemic mixture. To investigate the molecular mechanism of inhibition, we carried out docking runs with (*R*)- α-ionone, (*S*)- α-ionone, and β-ionone within the static initial ligand-free inactive PSGR model. As stated above, this is the structure that ionone would need to bind to according to the ternary complex model^48^. Figure 3b shows the respective best binding modes found. All three tested compounds are found in the same binding cavity, with their ionone ring positioned at the same position. The observed differences in binding result from the differences in stereochemistry at the connection between ring and butenoneyl side chain (in the following called “head group”). β-ionone and (*R*)- α-ionone bind in a very similar fashion with the head groups oriented towards Asp190^5.47^, while (*S*)- α-ionone binds with the head group oriented towards Lys185^5.42^. Both α-ionone enantiomers bind with a slightly higher affinity (∆G_bind_ = −6.4 kcal/mol and −6.9 kcal/mol) than β-ionone (∆G_bind_ = −6.1 kcal/mol). Furthermore, as free energy calculations performed on our MD trajectories (see Fig. 3c and Table SII) show that both α-ionone stereoisomers exhibit comparable and clearly separated affinities for the active and the inactive receptor conformation, with the affinity for the inactive conformation being significantly higher than the one for the active conformation. β-ionone exhibits an affinity for the receptor, which is comparable to the one of α-ionones for the inactive receptor, but is within the range of its standard deviation the same for the active and the inactive receptor. This result is in excellent agreement with the experimental observations: both α-ionones preferably bind to the inactive receptor conformation, and thus inhibit receptor activation. β-ionone exhibits a comparable affinity to both receptor conformations, and thus partially stabilizes the active conformation, leading to receptor activation. Due to the matching experimental and theoretical results, we assume that α-ionones bind to the same position as β-ionone, and act as orthosteric agonists opposed to a possible allosteric binding mode. In addition to this experiment, we investigated if our PSGR mutants exhibit a different response to co-application of *rac*- α-ionone and β-ionone. As Fig. 2b shows, while most mutants behave like WT receptors, we indeed observe an altered response for S107^3.36^V and K185^5.42^R: while S107^3.36^V activation receives a stronger block from *rac*- α-ionone than the WT, K185^5.42^R is less sensitive to the antagonist. This is a further indication that we have correctly identified the location of the odorant othosteric binding site within PSGR. In the following we will analyse the differences in dynamic binding modes of (*R*)- α-ionone, (*S*)- α-ionone and β-ionone in MD simulations to determine the molecular basis for ligand agonism/antagonism in PSGR.

### Dynamic binding modes define ligand effect on PSGR

**Figure 4:**
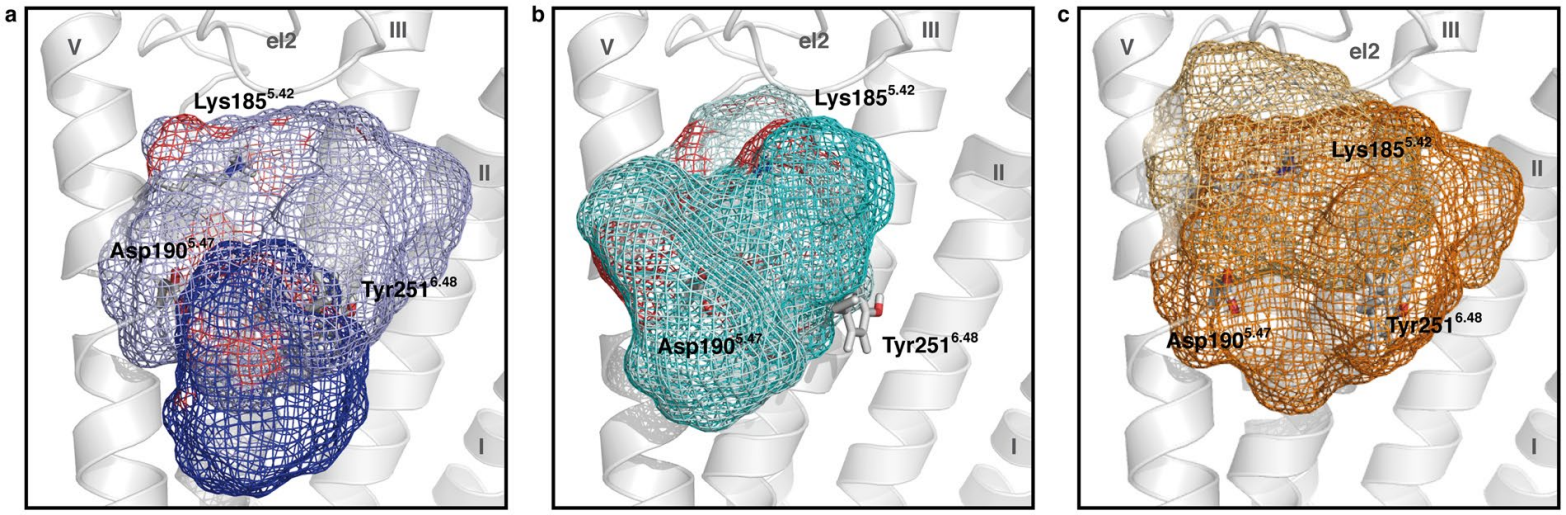
Selected ligand residence volumes during the last 10 ns of free MD simulation. Protein backbone in grey, selected surrounding protein residues in grey sticks. Helices numbered in roman numbers. (**a**) (*S*)- α-ionone. The dark blue mesh depicts the residence volume of (*S*)- α-ionone in the inactive state, and the light blue mesh depicts the residence volume in the active state. (**b**) (*R*)- α-ionone. The dark cyan mesh depicts the residence volume of (*S*)- α-ionone in the inactive state, and the light cyan mesh depicts the residence volume in the active state. (**c**) β-ionone. The dark orange mesh depicts the residence volume of β-ionone accessed in the inactive state model, and the light orange mesh the residence volume of β-ionone accessed in the active state model. The active and inactive state volumes of all ligands show a significant overlap. All ligands theoretically could transfer from their inactive state to their active state positions. Thus, binding position selectivity is no criterion for protein activation.

For a better sampling of ligand dynamics, we carried out three independent simulations with each ligand bound to the binding site as depicted in Fig. 3b. These ligand postures were generated by Docking as starting positions, but changed significant within the 40 ns equilibration phase of the respective models. Supplementary Figure S5 shows the resulting Cα-RMSD plots of the transmembrane helices for all simulation systems. All models stayed within a range of 2.5–3.5 Å in respect to the initial rhodopsin models. In our 50 ns runs of free MD simulations, we chose the last 10 ns for data assessment, as the protein models seemed structurally equilibrated and sufficiently stable during this period. Figure 4 depicts the ligand residence volumes of (*S*)- α-ionone, (*R*)- α-ionone, and β-ionone in both inactive and active model during these last 10 ns of MD simulation. We did not observe any specific binding position of ionones within the protein, as all three ligands were highly dynamic within the residence volumes. Furthermore, the comparison of binding positions of the correct ligand/protein model combinations (α-ionones in inactive protein conformation, β-ionone in active protein conformation) with the artificial residence volumes (α-ionones in active protein conformation, β-ionone in inactive protein conformation) shows that the residence volumes of all three ligands in both states exhibit a considerable overlap. Therefore, (*R*)- α-ionone, (*S*)- α-ionone and β-ionone should in theory be able to easily switch positions between active and inactive state – related positions. A difference in binding position alone cannot explain the selective activation of PSGR by β-ionone. In our earlier investigations, we found that ligand action on the protein is determined by distinct dynamic protein/ligand contacts (IFPs)^38,41^. In the following, we therefore analysed differences in such dynamic IFPs, and derived a model for the protein/protein and protein/ligand interactions relevant for protein activation. We here focus on the residues predicted by Docking to form part of a putative ligand binding site, and at the same time affect receptor activation upon mutation in experiments.

Table 2 displays dynamic protein/ligand contacts appearing in MD simulations with residues determined as crucial for protein activation via mutation analysis. Contacts were judged to be significant if they existed in at least two out of three simulation runs during >60% of simulated time. The following significant protein/ligand contacts were observed:

**Table 2:**
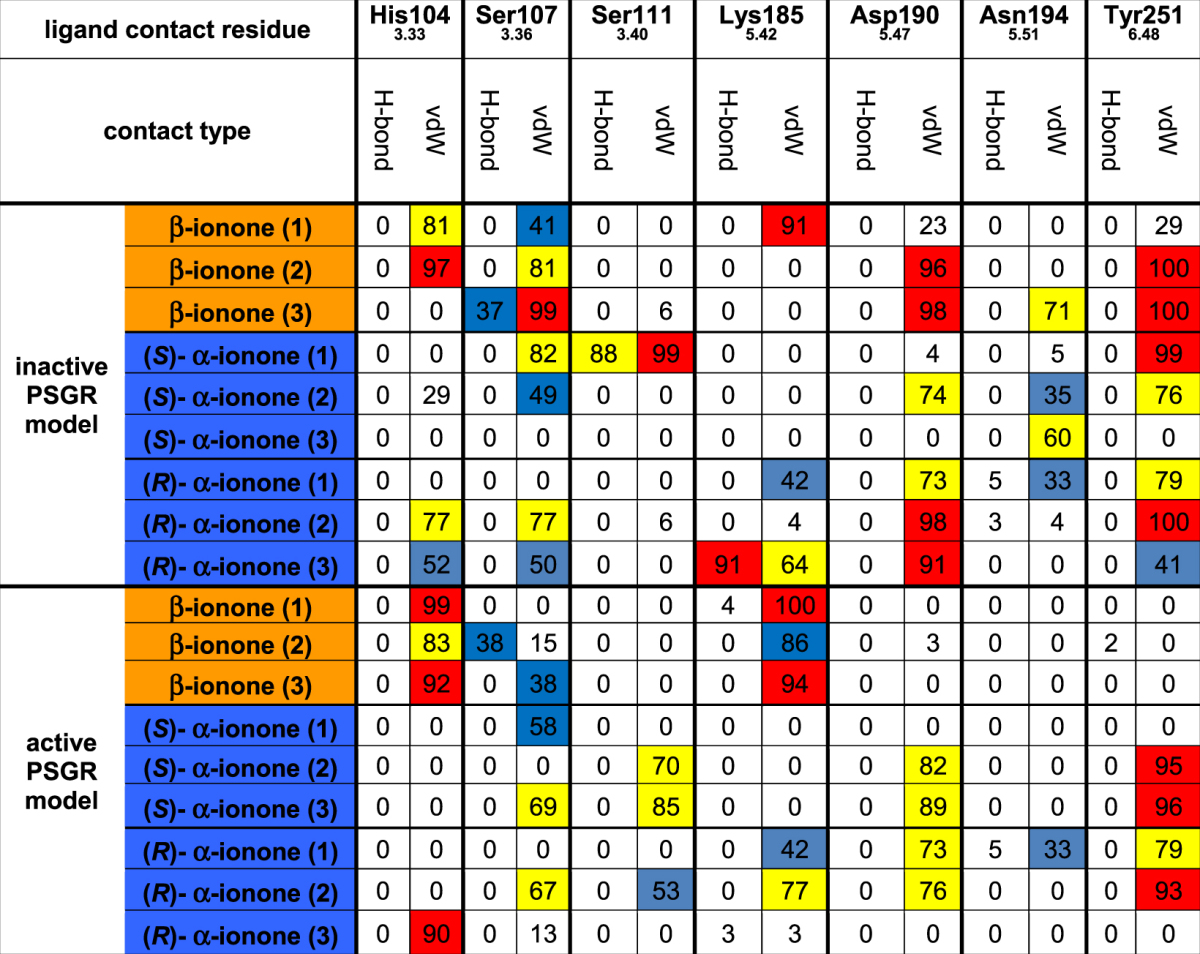
Dynamic protein/ligand contacts appearing in MD simulations. For improved sampling, three independent MD simulations were carried out with each ligand in each protein model. Contact types: H-bond: hydrogen bonding; vdW: van der Waals-contact. Colour coding: white: <30% contact occurrence during simulation; blue: contact present 30% to 60% of simulated time; yellow: contact present in 60% to 90% of simulated time; red: contact present in >90% of simulated time. Van der Waals contacts were counted as present if the distance between any atom of ligand and protein were within a distance that was equal or smaller than the sum of their van der Waals radii. Hydrogen bonds were counted as present if the distance between hydrogen bond donor and acceptor was 2.5–3.5 Å, and if the off-axis angle between O/N–H–O/N was smaller than 30°. Contacts were judged to be significant if they existed in at least two simulation runs during >60% of simulated time.

His104^3.33^: this residue only forms van der Waals-contacts with β-ionone, but not with α-ionones, in both active and inactive protein models. As the H104^3.33^F mutant is affected in its experimentally measured activity, His104^3.33^ seems to form part of the ligand cavity wall.

Ser107^3.36^: β-ionone forms significant contacts with this residue in the inactive state, but not in the active state. α-ionones do not exhibit any contacts with this residue. In experiment, mutation of Ser107^3.36^ to valine resulted in a hyperactive receptor. Therefore, Ser107^3.36^ might play a direct role in receptor activation by β-ionone. Concerning the increased efficiency of blockage by *rac*-α-ionone in S107^3.36^V, Table 2 shows that Ser107^3.36^ furthermore exhibits more frequent van der Waals contacts with α-ionones than with β-ionone. A hydrophobic mutation might increase the contact frequency with α-ionones, leading to better binding and thus blockage of β-ionone binding.

Ser111^3.40^: only a significant contact with (*S*)- α-ionone in the active state is observable. As (*S*)- α-ionone cannot activate the receptor, this is considered as an artificial contact. Nevertheless, Ser111^3.40^ was experimentally shown to be important for ligand-induced protein activation, as S111^3.40^V is an affected mutant (see Table 1).

Lys185^5.42^: no significant contacts are present for all ligands in the inactive conformation. In the active conformation, Lys185^5.42^ forms a significant van der Waals contact with β-ionone, but not with α-ionones. The experimentally observed affected activity/inactivity of the K185^5.42^L and K185^5.42^M mutants proves that this side chain is important for ligand-induced activation of PSGR. However, it further implies that the crucial feature of the Lys185^5.42^ side chain is its positive charge: a mutation retaining the charge distribution (K185^5.42^R) is still fully active. The K185^5.42^Q mutant, which does not exhibit a side chain charge, but still exhibits hydrogen bond capacity, is affected in its activity. Last, both K185^5.42^R and K185^5.42^Q exhibit a decreased basal activity, which further supports the involvement of the Lys185^5.42^ side chain in protein activation. Concerning the decreased efficiency of blockage by *rac*-α-ionone in K185^5.42^R, it might be possible that an intermediate distance charge interaction between the Lys185^5.42^ ammonium moiety and the α-ionone carbonyl atom can be formed in WT protein, similar to the situation in the Docking pose of (*S*)- α-ionone depicted in Fig. 3b, which is not present for β-ionone. The K185^5.42^R mutation therefore would affect (*S*)- α-ionone binding more than β-ionone binding.

Asp190^5.47^: this residue forms van der Waals contacts with β-ionone in the inactive state, but not in the active state, while (*S*)- α-ionone exhibits a contact pattern, which shows exactly the opposite behavior. (*R*)- α-ionone forms significant van der Waals contacts with this residue in both active and inactive state. Like in the case of Lys185^5.42^, the experimental mutant data implies that the side chain charge is the crucial feature for protein activation: while the D190^5.47^L mutant is affected in both ligand induced and basal activity, the D190^5.47^N mutant, which retains the possibility to form a hydrogen bond, is completely inactive.

Asn194^5.51^: as in the case for Ser111^3.40^, no significant protein/ligand contacts can be observed. However, as the N194^5.51^L mutant is experimentally inactive, Asn194^5.51^ has to be important for ligand-induced protein activation.

Tyr251^6.48^: in its inactive state, this residue forms van der Waals contacts with both α- and β-ionone. In the active state however, only a van der Waals contact with α-ionone is present. Like Ser107^3.36^, this residue therefore might play a significant role in protein/ligand activation. In agreement with this, in our sequence alignment used for creating the PSGR homology model, Tyr251^6.48^ is found at the position of Trp265^6.48^, the “rotameric toggle switch” in rhodopsin-like GPCRs^59^, which is crucial for the outward movement of helix VI, leading to G-protein binding and activation. A recent investigation found that this substitution of this tryptophane by a tyrosine is a general feature for the whole family of olfactory receptors^57^. However, the experimental mutant data shows that the hydrogen bonding capability of the tyrosine side chain seems to be the crucial determinant for ligand-induced protein activation: the Y251^6.48^F mutant is inactive.

From this list, it appears that the ligand does not form any side chain hydrogen bonds at all during simulation. As can be seen in Supplementary Table SII, even hydrogen bonds with the protein backbone are only observed for (*S*)- α-ionone in the active state, which is an artificial state. Selectivity of PSGR for ionones therefore seems to exclusively result from van der Waals contacts. However, it seems that van der Waals protein/ligand contacts can only explain the effect of the mutants of His104^3.33^ and Ser107^3.36^. The remaining mutants are either not easily explainable (Lys185^5.42^/Asp190^5.47^/Tyr251^6.48^) or not even coupled to protein/ligand contacts (Ser111^3.40^/Asn194^5.51^). It is possible that in the case of these residues, not only OR/ligand, but protein-internal contacts bonds are crucial for protein activation. In the following, we will therefore monitor the possible presence of protein/protein side chain interactions between these residues.

Table 3 displays such significant protein/protein contacts appearing during simulation. Judged by these data, (*R*)- α-, (*S*)- α- and β-ionone differ by the following protein/protein contacts:

**Table 3:**
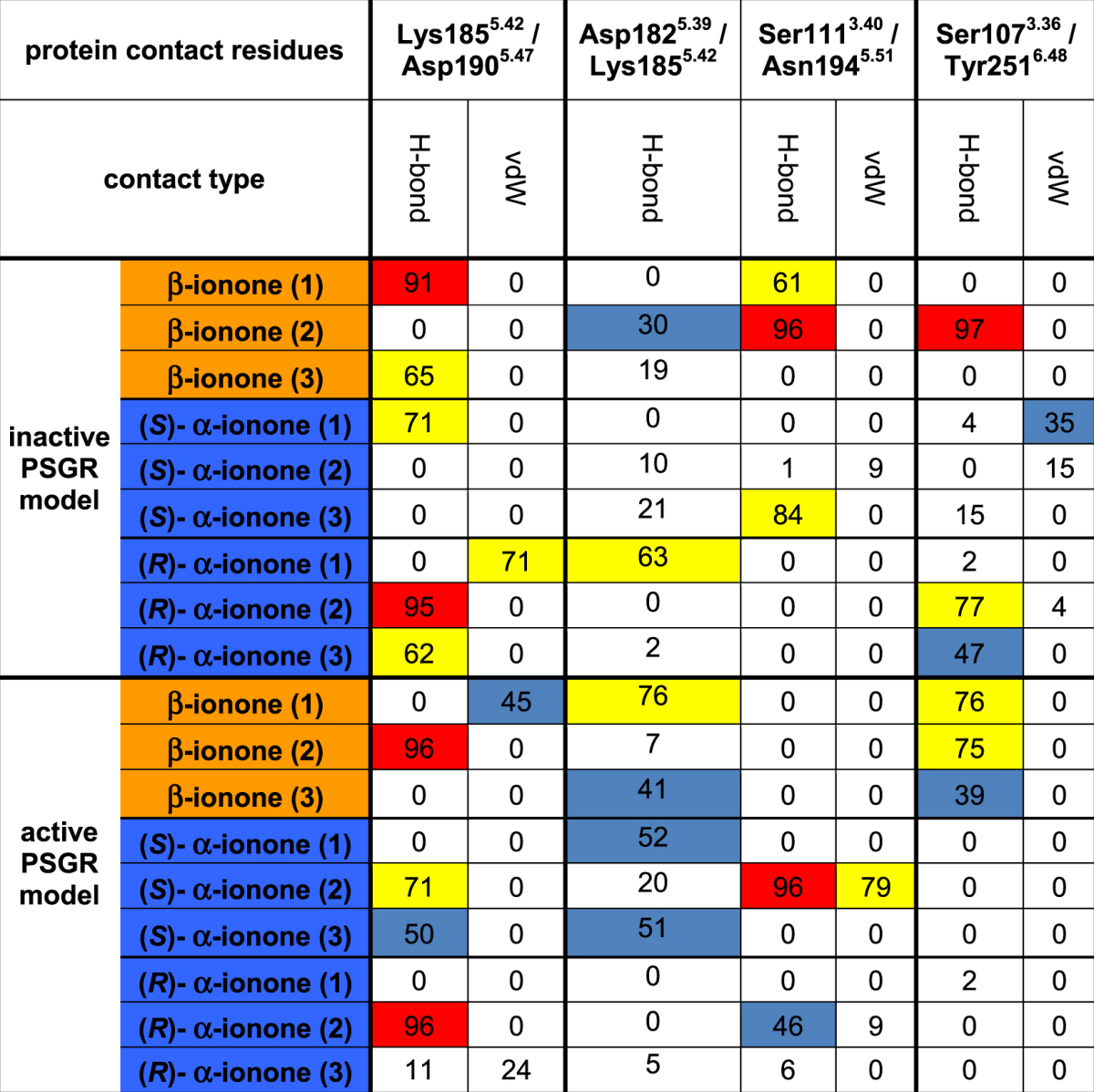
Dynamic protein/protein side chain contacts appearing in MD simulations. For improved sampling, three independent MD simulations were carried out with each ligand in each protein model. Contact types: H-bond: hydrogen bonding; vdW: van der Waals-contact. Colour coding: white: <30% contact occurrence during simulation; blue: contact present 30% to 60% of simulated time; yellow: contact present in 60% to 90% of simulated time; red: contact present in >90% of simulated time. Van der Waals contacts were counted as present if the distance between any atom of ligand and protein were within a distance that was equal or smaller than the sum of their van der Waals radii. Hydrogen bonds were counted as present if the distance between hydrogen bond donor and acceptor was 2.5–3.5 Å, and if the off-axis angle between O/N–H–O/N was smaller than 30°. Like in the case of protein/ligand contacts, contacts were judged to be significant if they existed in at least two simulation runs with >60% of simulated time.

Lys185^5.42^/Asp190^5.47^: both amino acids exhibit a salt bridge with β-ionone and (*R*)- α-ionone in the inactive state, but not in the active state. In simulations with (*S*)- α-ionone, this salt bridge is not significantly present. The presence of the salt bridge is in good agreement with our experimental mutant data: a mutation retaining the charge distribution (K185^5.42^R) is still fully active. Mutations quenching side chain charges, while still exhibiting hydrogen bond capacity (K185^5.42^Q, D190^5.47^N) are either affected or inactive. Lys185^5.42^ and Asp190^5.47^ therefore seem to be connected via a salt bridge, which needs to be formed in the inactive state, and to be broken by ligand binding to activate the receptor. This is in agreement with β-ionone exhibiting van der Waals contacts with Lys185^5.42^ in the active, but not in the inactive state. None of the (*S*)- α-ionone-bound models exhibits a Lys185^5.42^/ligand contact or the Lys185^5.42^/Asp190^5.47^ salt bridge, indicating a different protein side chain conformation distribution upon binding of this ligand. However, (*R*)- α-ionone behaves like β-ionone, allowing no clear discrimination of antagonism and agonism solely based on this intraprotein contact. We have to add here that the rupture of this salt bridge by an odorant would be energetically highly unfavourable. However, in simulations, we observe that the salt bridge is actually not broken, but shifted from Lys185^5.42^/Asp190^5.47^ to Asp182^5.39^/Lys185^5.42^ (see Table 3 and Figure S6), cancelling this enthalpic penalty.

Ser111^3.40^/Asn194^5.51^: similar to the case of Lys185^5.42^/Asp190^5.47^, Ser111^3.40^/Asn194^5.51^ exhibit a hydrogen bond, which exists in the inactive, β-ionone-bound state, while it is not significant in the active states and in simulations of α-ionones. The existence of this hydrogen bond in the inactive state and subsequent breakage of the bond seems therefore to be a prerequisite for receptor activation. Unlike in the case of the Lys185^5.42^/Asp190^5.47^ salt bridge, none of the two residues exhibits a significant van der Waals contact with any of the ligands. The alteration of the present hydrogen bond present here therefore seems to be an indirect effect of ligand binding.

Ser107^3.36^/Tyr251^6.48^: these two residues exhibit a hydrogen bond in the active, β-ionone bound state, which is not present in any other state. This coincides with both α-ionones and β-ionone forming a van der Waals contact with Tyr251^6.48^ in the inactive state, while in the active state, only (*S*)- α-ionone forms this contact. It therefore seems that formation of this hydrogen bond is crucial for protein activation, which is hindered by bound α-ionone molecules. This finding is in agreement with the observation that the “rotameric toggle” tyrosine in helix VI of olfactory receptors forms interactions with amino acid side chains in helix III^57^.

Furthermore, we checked if our investigated residues are found at evolutionary conserved or variable positions: residues conserved within the OR family are believed to be involved in receptor activation^57^, while hyper variable residues constitute to ligand selectivity^60^. We find that the interaction pair Ser107^3.36^/Tyr251^6.48^ was found to be a conserved contact motif crucial for receptor activation^57^, although position 3.36 is mostly occupied by a small hydrophobic residues, and that this contact is mostly a van der Waals contact. All other residues are highly variable throughout the OR family. Therefore, while ligand induced interruption of the Ser107^3.36^/Tyr251^6.48^ contact pair observed by us might be a conserved activation feature, the combination of all other residues forming the ligand binding site, and the resulting contact pairs Ser111^3.40^/Asn194^5.51^ and Lys185^5.42^/Asp190^5.47^ seem to be a specific feature of PSGR.

**Figure 5:**
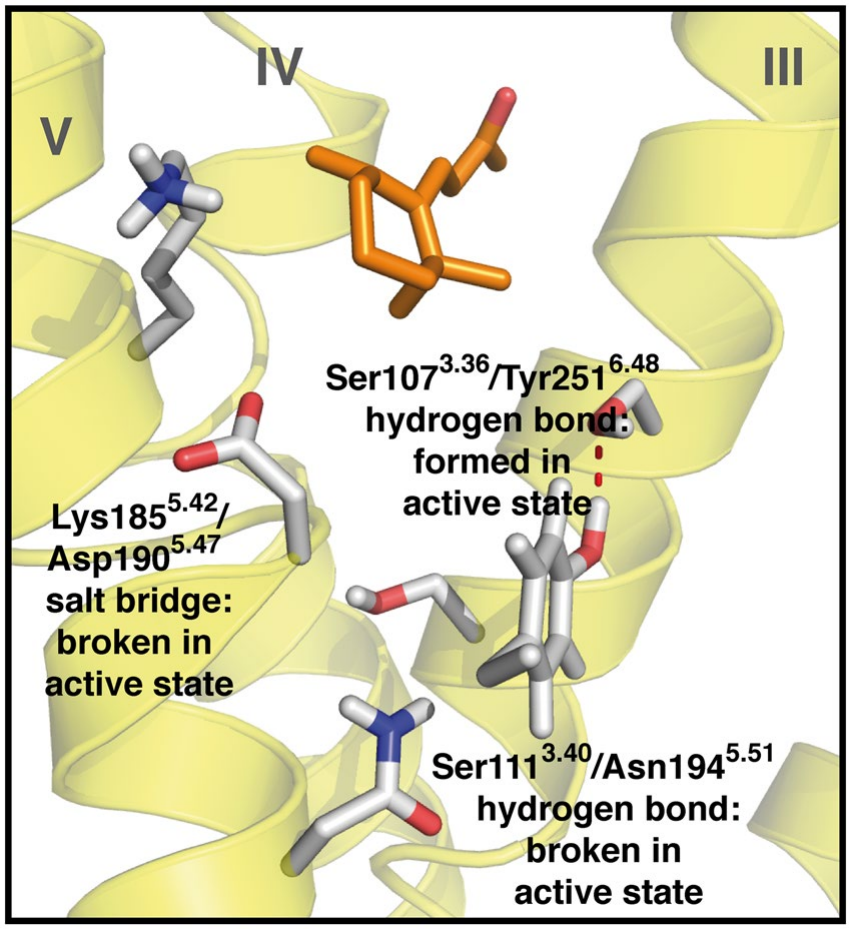
Proposed protein-internal hydrogen bond network in PSGR in the active state. The figure represents a snapshot out of the last 10 ns of free MD simulation (1^st^ β-ionone simulation in the active state listed in Table 2). The protein backbone in yellow, β-ionone in orange sticks, hydrogen bond network forming protein residues in grey sticks. Helices numbered in roman numbers. Upon protein activation, the Ser111^3.40^/Asn194^5.51^ hydrogen bond.and the Lys185^5.42^/Asp190^5.47^ salt bridge are broken. A new hydrogen bond is formed between Ser107^3.36^ and Tyr251^6.48^.

Summarizing these results, binding of α-ionones and β-ionone to PSGR seems to be coupled to the rearrangement of a protein-internal hydrogen bond network, which consists of the residues Ser107^3.36^, Ser111^3.40^, Lys185^5.42^, Asp190^5.47^, Asn194^5.51^, and Tyr251^6.48^, and is displayed in Fig. 5. This network links helices III (Ser107^3.36^, Ser111^3.40^) with V (Lys185^5.42^, Asp190^5.47^, Asn194^5.51^) and VI (Tyr251^6.48^), which are all known to change their relative positions upon receptor activation^61^. The modulation of this network is not achieved via hydrogen bond interaction between receptor and ligands, but via van der Waals interactions, which affect amino acid side chain conformations. Such a ligand-mediated modulation further offers an explanation for the weak effect of the K185^5.42^L and D190^5.47^L mutants, and the hyperactivity of the S107^3.36^V mutant: here, van der Waals contacts with the ligand might compensate for the missing crucial hydrogen bond connections listed above. Further support for this model comes from *in silico* data on changes of ligand binding affinities in a virtual alanine scanning we performed as displayed in Table 1: all investigated mutants, with the exception of I255^6.52^, exhibit a change in ligand affinity upon mutation to alanine *in silico* for all investigated ligands, which is in agreement with experiments on β-ionone, and further supported by the data on mutation effect on activation inhibition by α-ionones. The control mutant residue I255^6.52^ does not exhibit a significant influence on ligand binding. Interestingly, we find again that the best rationale between experiment and our theoretical data is provided by our hypothesis that disruption of intraprotein interaction partner connections by the ligand: The influence of mutation of Ser111^3.40^ cannot be explained by the impact of this residue on ligand binding affinities in the *in silico* alanine scanning. However, its interaction partner Asn194^5.51^ exhibits a significant impact on ligand binding, respectively. More pronounced even is this effect in the case of the Lys185^5.42^/Asp190^5.47^ pair: here, while Lys185^5.42^ only shows a weak influence on ligand binding, its interaction partner Asp190^5.47^ exhibits a strong influence.

## Discussion

Taking together our results so far, we see a good agreement between our theoretically predicted binding site model and the experimental point mutation analysis. Like in the case of our study on the olfactory receptor hOR2AG1^38^, receptor activation is achieved by modulation of the dynamics of a hydrogen bond system. However, in contrast to hOR2AG1, the respective hydrogen bond network in this case does not connect protein and ligand, but is completely protein-internal, connecting helices III, V, and VI. Especially the connection of helices III and VI via a “rotameric toggle” tyrosine is known to be an essential feature in olfactory receptor activation^57^. The ligands merely alter the connectivity via dynamic van der Waals interactions with amino acid side chains belonging to the hydrogen bond network. This mode of ligand-induced changes of protein-internal connections is in good agreement with the model that ligands alter the overall energetic landscape of possible protein conformations^62,63^. Interestingly, the two α-ionones do not induce an identical contact pattern: while they have the same effect on the interhelical hydrogen bonds of Ser107^3.36^/Tyr251^6.48^ and Ser111^3.40^/Asn194^5.51^, they differ in the effect on the hydrogen bond of Lys185^5.42^/Asp190^5.47^. We assume that this intrahelical salt bridge is less important for receptor activation than the interhelical hydrogen bonds, which are more likely to influence the packing and dynamics of the transmembrane helix bundle. Ligand recognition is highly stereo selective, as the investigated (*R*)- α-, (*S*)- α- and β-ionone majorly differ in the angle connecting the ionone ring and the butenoneyl side chain. To our surprise, we observe almost no hydrogen bonds to the keto moiety, which excludes this group as a possible pharmacophor for ligand recognition. At first glance, the absence of such a hydrogen bond seems energetically unfavourable, as it would require a desolvation of the keto moiety upon binding from the solvent. However, ionones are highly hydrophobic compounds, and therefore most likely do not enter the receptor from the solvent phase, but first enter the hydrophobic volume created by the membrane lipids, and then enter the protein from the membrane/protein interface, as it is the case with retinal in rhodopsin^64^. This cancels out the desolvation step during binding, removing this enthalpic penalty. It seems that the receptor simply does not form any hydrogen bond to the ligands, and relies on shape recognition via van der Waals interactions. This suggestion fits with the experimentally observed low affinity of ionones and PSGR, and the binding mode of β-ionone bound to rhodopsin in an x-ray structure (PDB ID 3OAX)^65^. The receptor therefore seems not to recognize the keto moiety as odotope^31–34^, but the whole shape of the ligands. While molecular models of OR/ligand interaction so far have mostly taken van der Waals contacts into account as elements of the binding site defining ligand binding affinity^33,38,39,50–52^, we here find them to be actively involved in receptor activation. At first glance, this result is in agreement with the recent findings of Baud *et al*.^40^, who used Docking on mOR-EG as major tool to assess receptor activation, and with comparisons of crystal structures of other classes of GPCRs in their active and inactive state^66,67^. However, we here find in our MD simulations that these van der Waals contacts are highly dynamic, and do not result in a singular stable ligand binding position. The resulting contact pattern seems to be specific enough to cause receptor activation only for β-ionone, but not for α-ionones. We do not expect this result to be an artefact from the modelling procedure, as our model can nicely explain the experimental data on amino acid mutations. We do not assume that the connection patterns found between PSGR and the tested ionones are representative for all ligands that possibly can activate PSGR. However, we believe that the general principle of activation, namely the modulation of protein-internal side chain contact frequencies, is valid for all olfactory GPCRs. We here have to state that in contrast to this low affinity, the detection threshold for β-ionone in the human nose is extremely low (<1ppb, i.e. <44 pM)^68^. It is well recognized that heterologous expression systems for ligand screening differ in composition of the signalling machinery compared to the native cellular environment. As such, our luciferase assay does not use the native olfactory signal cascade present in olfactory cilia. Consequently, the signalling efficacy might differ between our *in vitro* system and the human nose. Furthermore, we cannot exclude the missing presence of metal cofactors in the experiments^69^. However, PSGR signalling seems to depend on the cell type. PSGR in cancer cells does not signal via the native G_olf_, but hijacks different signalling pathways^18,19^ as well, which may account for the decreased sensitivity of these cells to β-ionone as well as different response kinetics compared to olfactory sensory neurons. While the receptor exhibits a low overall affinity for ionones and seemingly lacks a stable binding mode, it is highly stereo selective and can discriminate between the subtle structural differences of α-ionones and β-ionone.

In summary, we investigate the molecular interaction of the antagonist/agonist combination (*R*)- α-/ (*S*)- α-/ β-ionone on the ectopically expressed olfactory receptor PSGR, which is a potential anti-cancer drug target for prostate cancer, and especially the difficult to treat melanoma cancer. Based on our combined theoretical and experimental investigations, we suggest that this OR is activated by a ligand-induced rearrangement of a protein-internal hydrogen bond network. Like in our earlier investigations on hOR2AG1, this is achieved by the dynamic interaction between receptor and odorants^38^. Though the investigated ligands only exhibit a small geometric difference, the dynamic protein/ligand interaction pattern differs significantly. The network rearrangement is not performed by competing hydrogen bond interaction of the ligand with the network, but by van der Waals contacts of the ligand with the involved amino acid side chains, altering their conformations. Thus, the odotope recognition in this OR is highly stereo selective, but seemingly lacks any selectivity based on protein/ligand hydrogen bonds. In agreement with this, the experimentally observed affinity for β-ionone is very low, with threshold concentrations in the higher µM range. While the absence of a hydrogen bond between receptor and ionones seems surprising, binding of β-ionone to a GPCR (in this case: rhodopsin) has already been experimentally observed in an x-ray crystal structure^65^. As mentioned above, a further reason for the observed low affinity and efficacy might be the usage of a luciferase assay, which does not work via the native olfactory signal cascade. However, as this resembles the situation within prostate cancer cells^18,19^, the luciferase assay might be a valid model for signalling in ectopically expressed ORs. A future OR-based drug design scheme will have to be based on reproducing the correct dynamic protein/ligand IFPs^41^ instead of simply searching for complementarity of ligand and binding pocket shape. Furthermore, low-affinity ORs will need to be targeted with local high-dose drug applications.

## Methods

### Computational Methods

PSGR homology models were build by dynamic homology modelling^42^. The sequence of PSGR (Uniprot^70^ accession number Q9H255) was aligned to the one of rhodopsin (see Figure S1 for the alignment) with a focus on reproducing positions of the highest conserved helical residues according to Ballesteros-Weinstein numbering^55^. We chose rhodopsin as structural template, as is has been proven to be a good basis for modelling olfactory receptors^38,47^, and is the only GPCR that contains a hydrophobic ligand and is at the same time available as structural model in both an inactive and an active-like conformation. The model of PSGR in its inactive state was based on the inactive rhodopsin structure (PDB entry 1U19)^46^, while the model of active PSGR was based on the octylglycoside-bound active opsin structure (PDB ID 4J4Q)^47^. Ionone topologies and structures were obtained from the PRODRG server^71^. Ligand atomic charges were obtained from RESP charges from B3LYP/6-31 G* calculations^42^ in Gaussian09^72^. Docking was performed with Autodock Vina^43^, using a box of 20 × 20 × 20 Å^3^ with a grid spacing of 1 Å, covering the extracellular half of the protein. For conformational search, we kept the side chain single bonds in the ionones rotatable, and used an exhaustiveness factor of 80^41^. The most stable binding mode of each ligand (see Fig. 3b) was used as initial structure for simulation system creation. Protonation states were assigned with PROPKA^73^, with Asp69 and Glu110 being protonated. MD simulations were carried out with GROMACS (v4.0.5)^74^ and the GROMOS96 force field with Berger lipid parameters^75^ following the protocol of Wolf *et al*.^42^ with the receptor model being embedded in an explicit membrane (POPC)/solvent environment. Membrane insertion was performed with inflategro^76^. After addition of SPC water molecules, a steepest descent minimization with harmonic restraints on protein and ligand atomic coordinates (1000 kJ mol^−1^ nm^−2^) was followed by 5 ns of membrane/solvent equilibration with harmonic restraints on protein and ligand atomic coordinates (1000 kJ mol^−1^ nm^−2^). The full system was then energetically minimized using the steepest descent algorithm without any restraints. After velocity assignment for temperature generation (310 K), two consecutive steps of 100 ps MD simulations with stepwise reduced restraints (500 and 200 kJ mol^−1^ nm^−2^, respectively) followed, and free MD production runs without any restraints were carried out for 50 ns with 3 independent replica runs each (differing in their initial velocity distribution). We chose 50 ns as a suitable simulation length, as homology models with docked ligands quickly find into suitable binding poses^41,42^, but tend to diverge from a comparable crystal-structure based dynamic model: Figure S7 displays a short 10 ns MD simulation comparing the 7TM Cα RMSD of a beta(2) adrenergic receptor (B2AR) model build from a rhodopsin structure (PDB ID 1U19^46^) in comparison to a simulation of a beta(2) adrenergic receptor crystal structure (PDB ID 2RH1^77^). Already within this short time period, the RMSD quickly reaches 3.5 Å, which stands for a not reliable overall structure. Contrary to general belief, a MD simulation with a homology model therefore does not necessarily improve the overall structural model. This result might be discouraging concerning the usability of GPCR homology models in general. However, for the same example of a B2AR homology model, we found that when focussing on the binding site itself (ref.^41^, Figure S4), the model actually results in the right receptor/ligand contacts within 5 ns, and continues to exhibit this contact pattern for 95 ns. As summary of these two points, we judged that when using GPCR homology models to analyse protein/ligand interactions, it is more important to use repetitions of short trajectories instead of few long simulations. Taking the structural development of our PSGR models into account (see Figure S5), we took 50 ns simulation time as a good compromise. Replica runs were generated by the addition of different velocity distributions to each starting structure after the 2^nd^ minimisation. In each MD simulation period, the simulation system was held at 310 K with a Nose-Hoover thermostat (τ_T_ = 0.1 ps), and at a pressure of 1 bar with a Parinello-Rahman barostat (τ_P_ = 0.5 ps; semiisotropic coupling of membrane area axes and the transmembrane axis). As the transmembrane helix Cα-RMSD showed to be stable in all simulation systems during the last 10 ns of simulated time (see Figure S5), we used these 10 ns for data assessment. Residue contacts were evaluated with MOBY^78^. Ligand residence volumes were calculated based on snapshots taken each 0.1 ns during the assessment period of the last 10 ns from all replica runs. Free energy calculations were performed with GMXPBSA2.1^79,80^ As both α- and β-ionone are constitutional isomers with the same number and types of chemical bonds, the rotational and vibrational contribution to the overall entropy can be neglected while calculating relative and not absolute binding affinities. We needed to exclude two trajectories from the inactive state as outliers, as in these cases, the ligands spend a significant amount of time at the protein/membrane interface with parts of their structure, which led to an underestimation of the nonpolar ligand interaction energy (as no membrane is present in the energy evaluation process).

### Mutagenesis by Overlap Extension PCR and Cloning

Point mutations in PSGR were introduced at different positions using overlap extension PCR. Full-length rho1D4-tagged PSGR in pCI (Addgene, Cambridge, Massachusetts (MA), USA) served as a template. Initial PCRs provided mutated gene segments, with overlapping complementary 3′ ends carrying the desired point mutation, which were then mixed and used as a template for a subsequent PCR to generate the full-length product using flanking primers. Full-length primers included NotI and MluI restriction sites for further cloning into pCI (Promega, Madison, Wisconsin (WI), USA). The nucleotide sequence of the mutants was verified by sequencing. The primer pairs used for mutations are listed in the Supplementary Materials.

### Immunocytochemistry

Live-cell immunocytochemistry to evaluate cell-surface expression of PSGR variants was performed as described by Zhuang and Matsunami^81^ (see Supplementary Figure S2). Pictures were taken with Zeiss Axioskop 2 fluorescence microscope and Axiovision software (Zeiss, Jena, Germany).

### Luciferase assay

The Dual-Glo Luciferase Assay System (Promega, Madison, Wisconsin, USA) was used to measure cellular responses as an indirect measure of receptor activation as previously described. It is the most commonly used method for high-throughput screening of odorant-receptor pairs^81,82^. This method quantifies cellular responses as an indirect measure of odorant receptor activation Hana3A cells seeded on a 96-well plate (NUNC, Thermo Fisher Scientific, Waltham, Massachusetts (MA), USA) were transfected with Lipofectamine 2000 (Invitrogen, Invitogen, Carlsbad, California (CA), USA) according to the manufacturer’s protocol using 18 µl Lipofectamine, 1 µg of RTP1S plasmid^83^, 1 µg of pRL-TK-*Renilla* (Promega), 2 µg of pGL4.29-luciferase (Promega), 1 µg of hM3^84^ and 5 µg of plasmids encoding for olfactory receptors for an entire well plate. 18–24 h after transfection, transfection medium was removed and replaced with the appropriate concentration of odorant, 0.1% DMSO (negative control) or 10 µM forskolin (positive control) in CD293 medium (Gibco; Life Technologies, Carlsbad, CA, USA) with 2 mM L-glutamine. Ultrapure β-ionone was a generous gift of Dr. J. Panten (Symrise, Holzminden, Germany). *rac*-α-ionone was purchased from Sigma-Aldrich. Odorant stocks were diluted in DMSO (Sigma–Aldrich, Munich, Germany). Four hours after odor stimulation, luminescence was measured using the microplate reader Fusion (Packard BioScience, Meriden, Connecticut (CT), USA). Firefly luminescence values were divided by the *Renilla* luciferase activity to control for transfection efficiency in a given well. The firefly-*Renilla* luciferase ratio was normalized against the lowest/highest luciferase ratios obtained for that experiment. Normalized luciferase activity was calculated by the formula (Luc/Ren(N) − Luc/Ren(lowest))/(Luc/Ren(highest) − Luc/Ren(lowest), where Luc/Ren(N) is the luminescence of firefly luciferase divided by luminescence of *Renilla* luciferase in a certain well; Luc/Renilla(lowest) is the lowest luciferase ratio of PSGR mutants transfected cells to negative control; Luc/Ren(highest) is the maximum luciferase ratio of PSGR mutant- transfected cells to the positive control of a plate. Mock-transfected cells were stimulated to exclude unspecific responses to the tested compounds. The dose response curves were fitted by *Hill* equation. The threshold concentration (10% signalling output level of 10 µM forskolin) was determined using dose response curve. The luciferase value of receptor mutants after stimulation with 250 µM β-ionone (highest applicable odorant concentration) was normalized to the value of wild type receptor to calculate the E_max_ value. Data were analysed with Microsoft Excel and SigmaPlot.

### Calcium imaging

HEK293 cells were grown in 35-mm cell culture dishes (50% confluence) and transfected with pcDNA3-PSGR (1 µg) using a standard calcium-phosphate precipitation technique. After 48 hours cells were incubated for 30 minutes at 37 °C with Ringer’s solution (140 mM NaCl, 5 mM KCl, 10 mM HEPES, 2 mM CaCl_2_, 1 mM MgCl_2_; pH 7.4) and 3 µM Fura-2-AM (Life Technologies, Carlsbad, California, USA). Calcium imaging experiments were performed as described in Spehr *et al*.^5^ and Neuhaus *et al*.^2^.

## Electronic supplementary material

Supplementary Information
