# Supplementary material for: Dynamical Binding Modes Determine Agonistic and Antagonistic Ligand Effects in the Prostate-Specific G-Protein Coupled Receptor (PSGR)
Source: Sci Rep. 2017 Nov 22;7:16007. doi: 10.1038/s41598-017-16001-4 (PMC5700038; doi:10.1038/s41598-017-16001-4)
Supplement: Supplementary file 1 — Supplementary Information [file 41598_2017_16001_MOESM1_ESM.pdf]

Dynamical Binding Modes Determine Agonistic and Antagonistic Ligand Effects in the Prostate-Specific G-Protein Coupled Receptor (PSGR)

Steffen Wolf, Nikolina Jovancevic, Lian Gelis, Sebastian Pietsch, Hanns Hatt, and Klaus Gerwert

Supplementary Information

Primer pairs used for mutations:

Flanking primers:

gcatatacgcgtatgagttcctgcaacttc (forward)

gcatatgcggccgctcacttgcctcccacagc (reverse)

Internal primers:

H104F

gttctttatttttgccctctcagccattgaatc (forward)

ctgagagggcaaaaataaagaacatctgggtaag (reverse)

S107V

catgccctcgtagccattgaatccaccatcctgc (forward)

gattcaatggctacgagggcatgaataaagaac (reverse)

S111V

gccattgaagtcaccatcctgctggccatgg (forward)

gcaggatggtgacttcaatggctgagagggcatg (reverse)

K185L

ggatgtaatgctgttggcctatgcagacactttgc (forward)

gcataggccaacagcattacatcctggtggacac (reverse)

K185R

ggatgtaatgcggttggcctatgcagacactttgc (forward)

gcataggccaaccgcattacatcctggtggacac (reverse)

K185Q

ggatgtaatgcagttggcctatgcagacactttgc (forward)

gcataggccaactgcattacatcctggtggacac (reverse)

K185M

ggatgtaatgatgttggcctatgcagacactttgc (forward)

gcataggccaacatcattacatcctggtggacac (reverse)

A189I

aatgaagttgatctatgcagacactttgcccaatg (forward)

gtctgcatagatcaacttcattacatcctggtgg (reverse)

D190L

ggcctatgcactcactttgcccaatgtggtatatg (forward)

gggcaaagtgagtgcataggccaacttcattac (reverse)

D190N

ggcctatgcaaacactttgcccaatgtggtatatg (forward)

gggcaaagtgtttgcataggccaacttcattac (reverse)

N194L

cactttgcccttagtggtatatggtcttactgc (forward)

catataccactaagggcaaagtgtctgcatagg (reverse)

Y251F

actcgccttctttgtgccacttattggcctctc (forward)

aagtggcacaaagaaggcgagtaccacaccaatg (reverse)

I255W

gtgccactttggggcctctcagtggtacacc (forward)

ctgagaggccccaaagtggcacatagaaggcgag (reverse)

Supplementary Figures

```
                                                                I
Rhod    1 MNGTEGPNFYVPFSNKTGVVRSPFEAPQYYLAEPWQFSMLAAYMFLLIMLGFPINFLTLYVTVQ 64
                 NF G                                           N
51E2    1 ---MSSCNFTHATFVLIGIPGL-----------EKAHFWVGFPLLSMYVVAMFGNCIVVFIVRT 50

                              II                                  III
Rhod   65 HKKLRTPLNYILLNLAVADLFMVFGGFTTTLYTSLHGYFVFGPTGCNLEGFFATLGGEIALWS 127
          ++ L+ P    L  LA  DL          L              C    FF      I
51E2   51 ERSLHAPMYLFLCMLAAIDLALSTSTMPKILALFWFDSREISFEACLTQMFFIHALSAIESTI 113

                                            IV
Rhod  128 LVVLAIERYVVVCKPMSNFRFGENHAIMGVAFTWVMALACAAPPLVGWSRYIPEGMQCSCGID 190
          L   A +RYV  C+P       +N          V        PL    +         C  +
51E2  114 LLAMAFDRYVAICHPLRHAAVLNNTVTAQIGIVAVVRGSLFFFPLPLLIKRLAF-----CHSN 171

                                V
Rhod  191 YYTPHEETNNESFVIYMFVVHFIIPLIVIFFCYGQLVFTVKEAAAQQQESAT--------TQK 245
                                  P  V       LV  V+
51E2  172 VLSHSY---CVHQDVMKLAYADTLPNVVYGLTAILLVMGVDVMFISLSYFLIIRTVLQLPSKS 231

                       VI                                        VII
Rhod  246 AEKEVTRMVIIMVIAFLICWLPYAGVAFYIFTHQGSDFGPIFMTIPAFFAKTSAVYNPVIYIM 308
           + +            L    P  G          S                  V NP IY
51E2  232 ERAKAFGTCVSHIGVVLAFYVPLIGLSVVHR-FGNSLHPIVRVVMGDIYLLLPPVINPIIYGA 293

                    VIII
Rhod  309 MNKQFRNCMVTTLCCG 324
            KQ R
51E2  294 KTKQIRTRVLAMFKIS 309
```

Figure S1. Sequence alignment used for homology model building. Transmembrane helices in rhodopsin highlighted in red, highest conserved helical residues[3] highlighted in yellow.

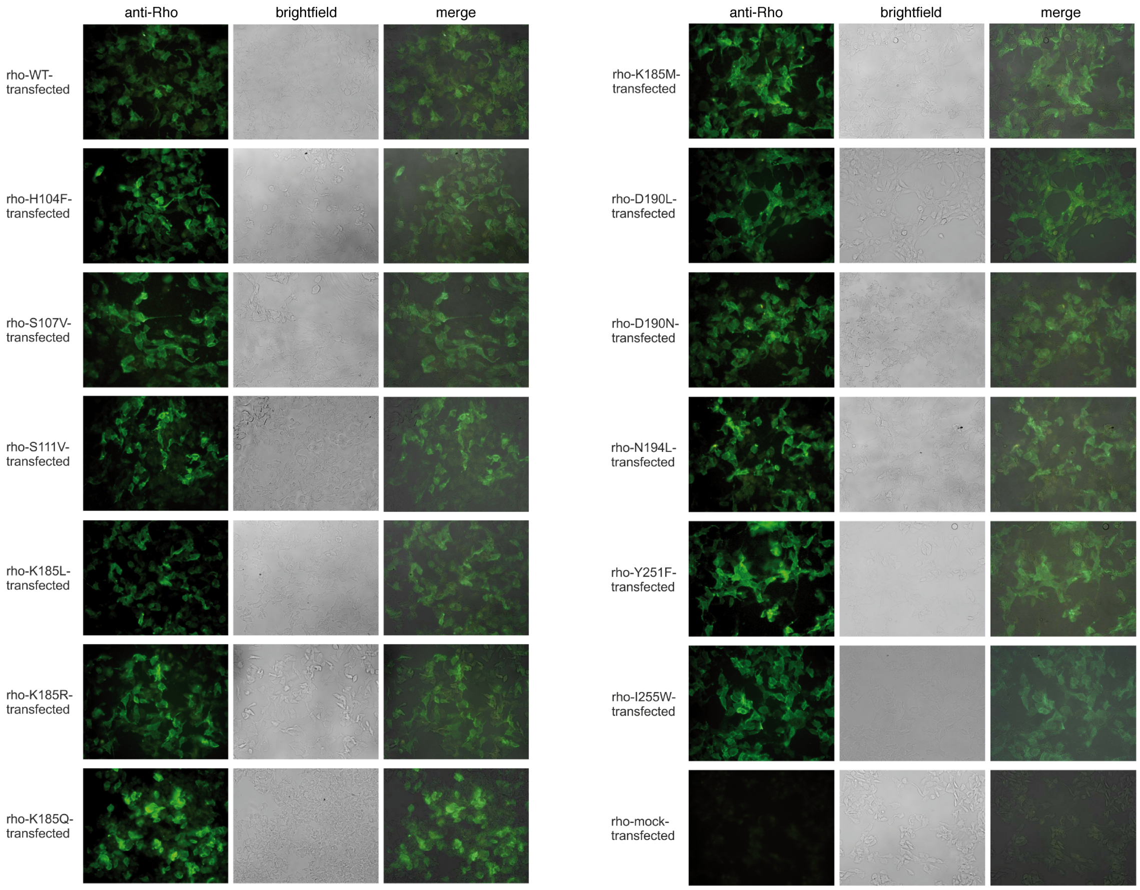

Figure S2. Cell-surface expression of wild type and mutant PSGR. Heterologous expression of rhodopsin-tagged wild type (WT) and point- mutated PSGR in transfected Hana3A cells was detected by the anti-rhodopsin antibody 4D2 and a secondary antibody labelled with the fluorescent dye Alexa Fluor 488 (green). Mock-transfected Hana3A cells served as negative control.

a

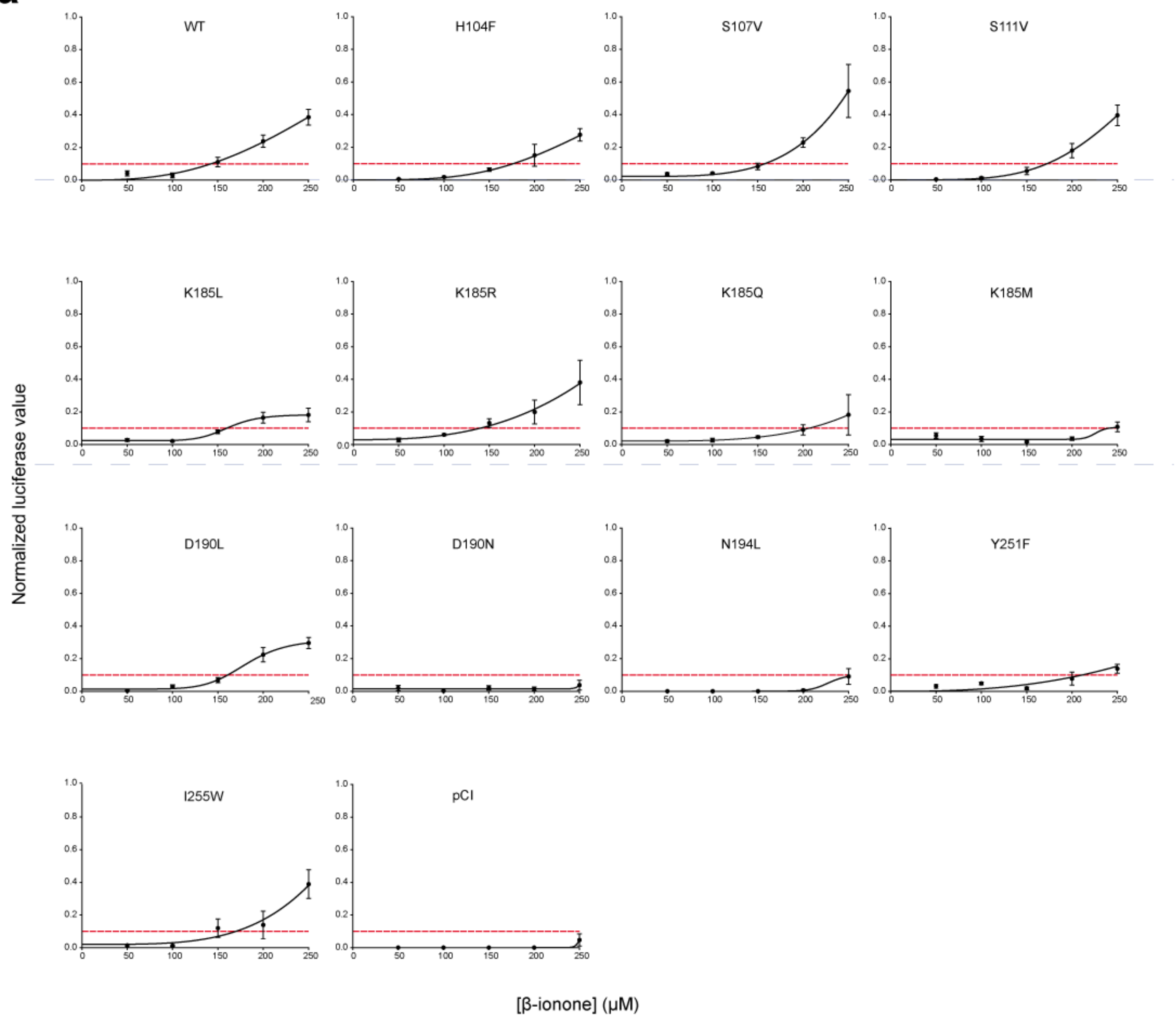

b

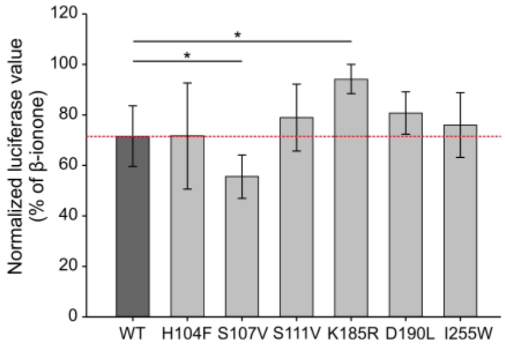

Figure S3. Dose-response curves of PSGR variants. (a) Responses of cells transfected either with a plasmid encoding for wild type (WT) and point-mutated PSGR, respectively, or the empty vector (pCI) to β-ionone as measured by luciferase assay. Receptor activation was

normalized to the response to forskolin (10 μM). Dose-response curves were fitted by a *Hill* equation. Odorant concentrations up to 250 μM were used because higher concentrations of **β**-ionone exhibited toxic effects on the cells as cells detached during treatment. Red dashed line represents the response threshold (set at 10% forskolin total response). Error bars indicate the standard error of the mean (SEM) of 4-15 replicates. (b) Quantification of luciferase values of Hana3a cells transiently expressing wild type (WT) or point-mutated PSGR co-stimulated with β-ionone (200 μM) and α-ionone (200 μM) or β -ionone (200 μM) only. The blocking effect of α -ionone was normalized to the corresponding response to β -ionone of the respective receptor. The data are shown as the means ± SEM (n=3). Significance was calculated by Student's t-test (*: $p<0.05$).

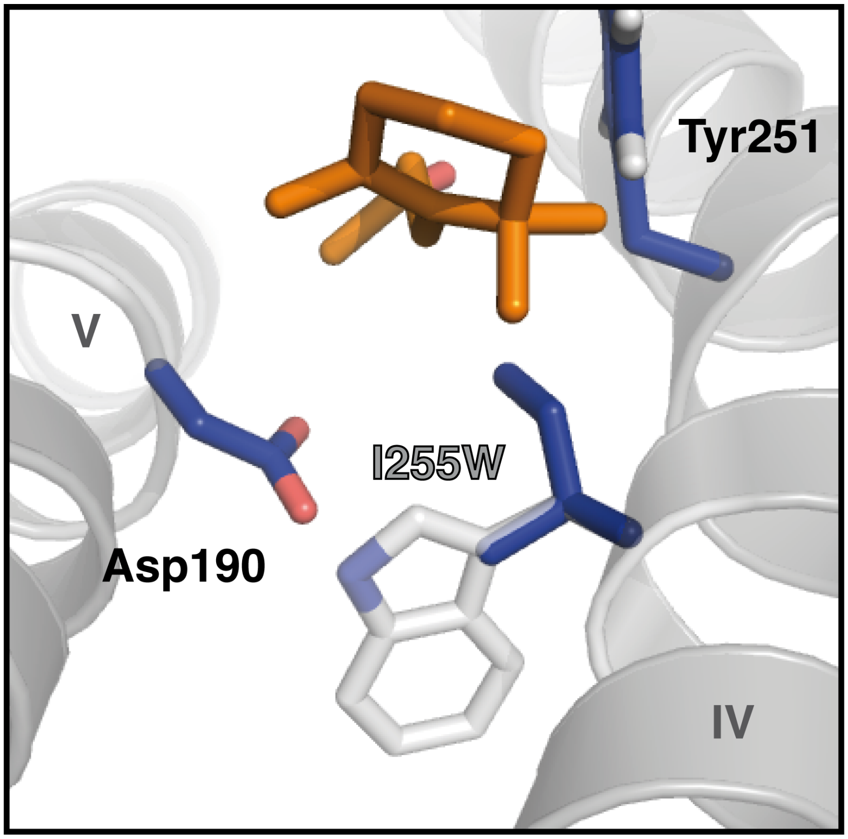

Figure S4. Position and influence of control mutant I255W. Protein backbone in grey, β-ionone in orange sticks, selected surrounding protein residues as blue sticks, mutation as transparent grey sticks. Helices numbered in roman numbers. The I255W mutant has sufficient space to place the size-increased side chains without occupying the ionone binding site or interfering with the protein itself. This is in agreement with the experimental observation that both mutants retain activity.

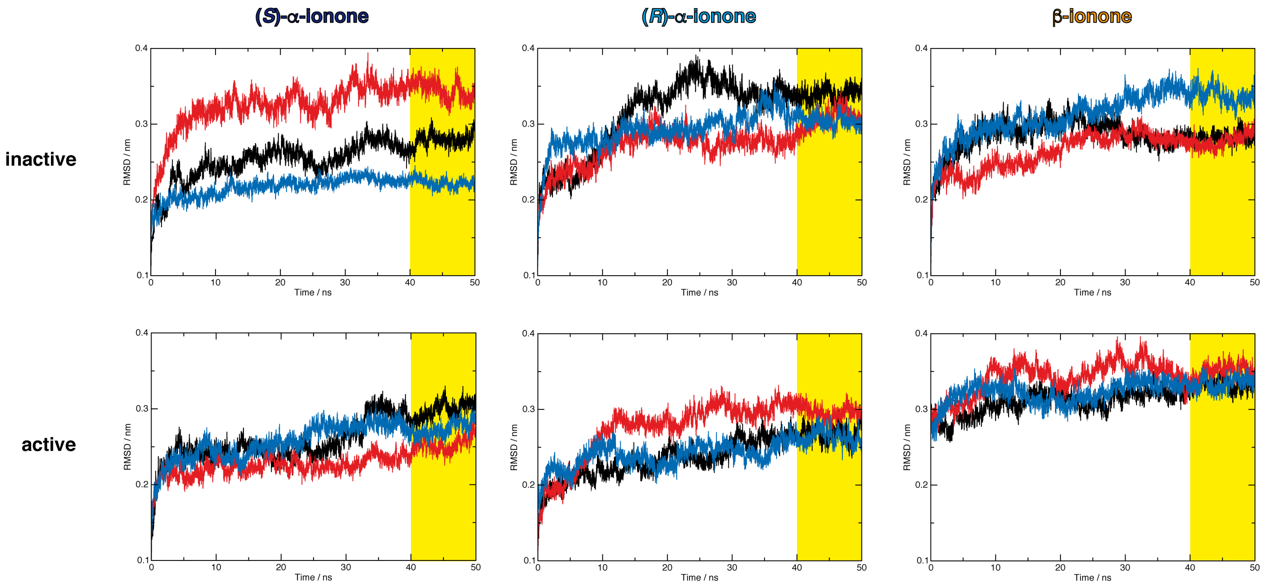

Figure S5. Transmembrane helix C α-RMSD of simulation systems listed in Tables I, II, and SI. System (1) in black, (2) in red, and (3) in blue. Last 10 ns highlighted in yellow. All models stay within a RMSD range of 2.5 to 3.5 Å and are structurally stable within the last 10 ns, which were used for binding mode analysis.

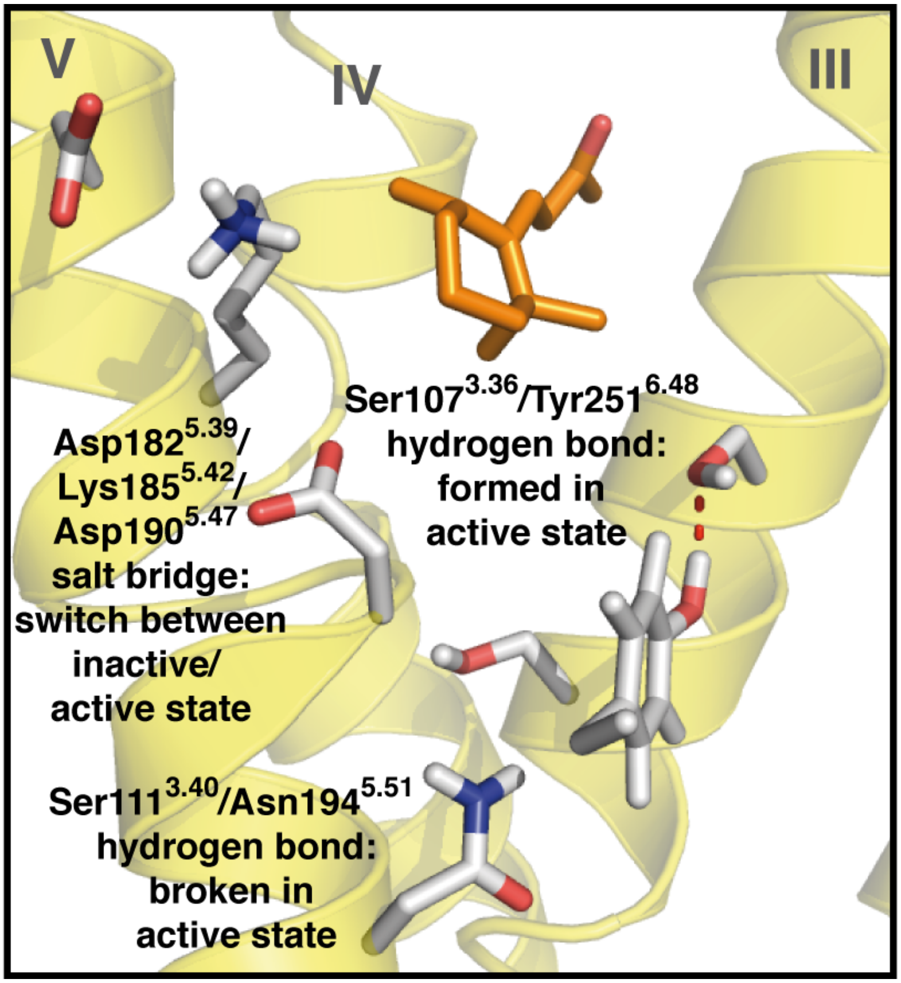

Figure S6. Extended view on the proposed protein-internal hydrogen bond network in PSGR in the active state. The figure represents a snapshot out of the last 10 ns of free MD simulation (1st β-ionone simulation in the active state listed in Table IV). The protein backbone in yellow, β-ionone in orange sticks, hydrogen bond network forming protein residues in grey sticks. Helices numbered in roman numbers. Additionally to the changes proposed in Figure 5, the breaking of the Lys185$^{5.42}$/Asp190$^{5.47}$ salt bridge is energetically balanced by the formation of a new salt bridge between Asp182$^{5.39}$ and Lys185$^{5.42}$.

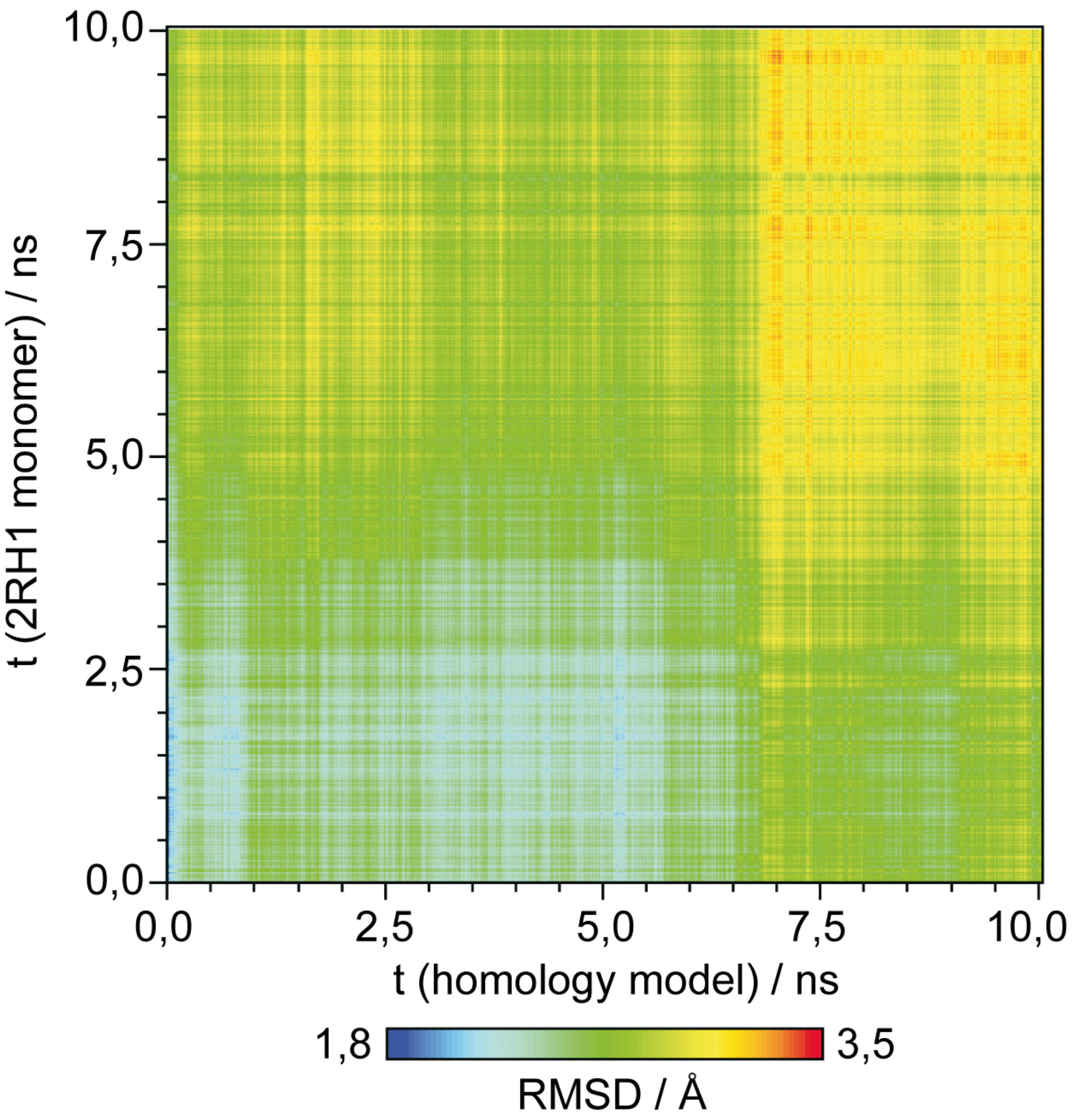

Figure S7: Analysis of a short 10 ns MD simulation comparing the 7TM C(alpha) RMSD of a beta(2) adrenergic receptor (B2AR) model build from a rhodopsin structure (PDB ID 1U19) (x axis) to a simulation of a beta(2) adrenergic receptor crystal structure (PDB ID 2RH1) (y axis). Already within this short time period, the RMSD quickly reaches 3.5 Å, which stands for a not reliable overall structure. Contrary to general belief, a MD simulation with a homology model therefore does not necessarily improve the overall structural model.

Supplementary Tables

Table SI. Effects of mutation on basal activity level. The basal activity level of cells transfected either with a plasmid encoding for WT and point-mutated PSGR, respectively, was measured by luciferase assay. The data are shown as the means ± SEM (n=3-4). Significance was calculated by Student's t-test (*: p<0.05; ns: not significant).

protein	basal activity level	p-value
WT	23.0±4.4	(reference)
H104$^{3.33}$F	4.9±0.1	ns
S107$^{3.36}$V	10.8±5.3	ns
S111$^{3.40}$V	8.8±3.9	ns
K185$^{5.42}$L	12.2±2.0	ns
K185$^{5.42}$R	4.5±1.2	*
K185$^{5.42}$Q	9.0±1.7	*
K185$^{5.42}$M	11.1±3.8	ns
D190$^{5.47}$L	3.3±0.2	*
D190$^{5.47}$N	11.1±2.4	ns
N194$^{5.51}$L	12.0±2.0	ns
Y251$^{6.48}$F	16.3±3.4	ns
I255$^{6.52}$W	42.8±24.5	ns

Table SII. Hydrogen bond contacts of ligands with the protein backbone. For improved sampling, three independent MD simulations were carried out with each ligand in each protein model. Colour coding: white: <30% contact occurrence; blue: contact present 30% to 60% of simulated time; yellow: contact present in 60% to 90% of simulated time; red: contact present in >90% of simulated time. Hydrogen bonds were counted as present if the distance between hydrogen bond donor and acceptor was 2.5–3.5 Å, and if the angle between O/N–H–O/N was smaller than 30°. Contacts were judged to be significant if they existed in at least two simulation runs with >60% of simulated time.

ligand contact residues		Gly18	Ala22	Arg89	Cys168	Val172	Leu173	Ser174
inactive PSGR model	β-ionone (1)	0	0	0	0	0	0	0
	β-ionone (2)	0	0	0	0	0	0	0
	β-ionone (3)	0	0	0	0	0	0	0
	(*S*)- α-ionone (1)	0	97	0	0	0	0	0
	(*S*)- α-ionone (2)	67	0	0	90	0	0	0
	(*S*)- α -ionone (3)	0	0	2	0	0	0	0
	(*R*)- α-ionone	0	0	0	0	0	0	0
	(*R*)- α-ionone (2)	0	0	0	0	0	0	0
	(*R*)- α-ionone (3)	0	0	0	0	0	0	0
active PSGR model	β-ionone (1)	0	0	0	0	76	45	0
	β-ionone (2)	0	0	0	0	0	0	0
	β-ionone (3)	0	0	0	0	0	0	0
	(*S*)- α-ionone (1)	0	0	0	0	99	99	0
	(*S*)- α-ionone (2)	0	0	0	0	83	74	44
	(*S*)- α-ionone (3)	0	0	0	0	0	98	0
	(*R*)- α-ionone (1)	0	0	0	0	0	0	0
	(*R*)- α-ionone (2)	0	0	0	0	0	0	0
	(*R*)- α-ionone (3)	0	0	0	0	0	0	0

Table SIII. Free energies of binding in kcal/mol.

	run #	outlier?[1]	$\Delta G_{MM\text{-}GBSA}$	STD[2]	$<\Delta\Delta G_{bind}>$[3,4]	STD
**inactive**						
**(*S*)-α-ionone**	1		-29.8	0.2	**1.7**	**1.2**
	2		-27.4	0.1		
	3	**-outlier-**	-11.9	0.1		
**(*R*)-α-ionone**	1		-28.5	0.1	**1.5**	**0.5**
	2		-28.3	0.1		
	3		-29.4	0.1		
**β-ionone**	1	**-outlier-**	-16.9	0.1	**0.0**	**1.7**
	2		-28.6	0.1		
	3		-31.9	0.1		
**active**						
**(*S*)-α-ionone**	1		-22.1	0.1	**8.1**	**0.3**
	2		-22.6	0.1		
	3		-21.8	0.1		
**(*R*)-α-ionone**	1		-22.6	0.1	**6.7**	**2.1**
	2		-26.4	0.1		
	3		-21.5	0.1		
**β-ionone**	1		-33.9	0.1	**1.4**	**3.6**
	2		-25.9	0.1		
	3		-26.8	0.1		

[1]if the ligand is partially positioned at the protein/membrane interface (results in underestimated nonpolar interaction)
[2]standard deviation
[3]with $<\Delta G_{bind}> \approx <\Delta G_{MM\text{-}GBSA}>$ due to neglectable differences in entropy between ligands
[4]with $<\Delta G_{bind}>$ of β-ionone bound to the inactive receptor as reference
